# Patterning Techniques Based on Metallized Electrospun Nanofibers for Advanced Stretchable Electronics

**DOI:** 10.1002/advs.202309735

**Published:** 2024-04-30

**Authors:** Yuhan Bian, Haozhou Shi, Qunchen Yuan, Yuxuan Zhu, Zhengzi Lin, Liujing Zhuang, Xun Han, Ping Wang, Mengxiao Chen, Xiandi Wang

**Affiliations:** ^1^ Department of Biomedical Engineering, Key Laboratory for Biomedical Engineering of Education Ministry, Zhejiang Provincial Key Laboratory of Cardio‐Cerebral Vascular Detection Technology and Medicinal Effectiveness Appraisal Zhejiang University Hangzhou 310027 P. R. China; ^2^ ZJU‐Hangzhou Global Scientific and Technological Innovation Center School of Micro‐Nano Electronics Zhejiang University Hangzhou 311200 P. R. China; ^3^ Research Center for Humanoid Sensing Zhejiang Lab Hangzhou 311121 P. R. China

**Keywords:** electrospun nanofiber, metalized nanofibers, patterning techniques, stretchable electronics

## Abstract

Stretchable electronics have experienced remarkable progress, especially in sensors and wireless communication systems, attributed to their ability to conformably contact with rough or uneven surfaces. However, the development of complex, multifunctional, and high‐precision stretchable electronics faces substantial challenges, including instability at rigid‐soft interfaces and incompatibility with traditional high‐precision patterning technologies. Metallized electrospun nanofibers emerge as a promising conductive filler, offering exceptional stretchability, electrical conductivity, transparency, and compatibility with existing patterning technologies. Here, this review focuses on the fundamental properties, preparation processes, patterning technologies, and application scenarios of conductive stretchable composites based on metallized nanofibers. Initially, it introduces the fabrication processes of metallized electrospun nanofibers and their advantages over alternative materials. It then highlights recent progress in patterning technologies, including collector collection, vapor deposition with masks, and lithography, emphasizing their role in enhancing precision and integration. Furthermore, the review shows the broad applicability and potential influence of metallized electrospun nanofibers in various fields through their use in sensors, wireless systems, semiconductor devices, and intelligent healthcare solutions. Ultimately, this review seeks to spark further innovation and address the prevailing challenges in stretchable electronics, paving the way for future breakthroughs in this dynamic field.

## Graphical abstract

Patterning techniques of stretchable materials plays an important role in the development of high‐precision stretchable electronics. Especially, metallized electrospun nanofibers as the conductive filler show excellent comprehensive abilities, including high stretchability, low percolation threshold, and compatibility with traditional patterning technologies. In this review, it will be introduced in detail on the relevant theoretical basis, preparation process and application prospect.

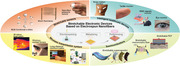

## 1 Introduction

Stretchable electronics, an extension of flexible electronics, presents a solution with its remarkable capacity to stretch, compress, twist, and conform into intricate, curvilinear shapes. Recent advancements have demonstrated various successful applications in laboratory settings, spanning stretchable sensors, wireless transmission systems, semiconductor devices, and intelligent healthcare systems. These breakthroughs lay the groundwork for the future industrialization of next‐generation electronics.^[^ ^1^ ^]^

In the development of stretchable electronics, a combination of structure optimization and advanced material development has been instrumental. Integrating conventional high‐performance electronic components with elastic substrates has enabled the fabrication of a diverse range of high‐performance stretchable devices. Structural optimization techniques, such as the island‐bridge design and mesh or crack design, have been employed to enhance device performance.^[^ ^2^ ^]^ However, a significant challenge faced by these devices lies in packaging, as the Young's modulus at rigid‐soft interfaces often does not align seamlessly. To address this challenge, novel intrinsically stretchable materials, including stretchable conductors, semiconductors, and insulators, are being explored as essential elements in the development of highly integrated stretchable electronics. These materials offer the potential to overcome packaging issues and facilitate the creation of more seamless and robust stretchable electronic systems.^[^ ^3^ ^]^

Recently, new functional materials, such as conductive composites based on percolation theory, high‐molecular conductive polymers, and ionic conductors,^[^ ^4^ ^]^ have shown promise for multifunctional stretchable electronics. To meet the evolving demands of miniaturization, wireless portability, and multifunctionality in electronics, stretchable conductors must not only possess excellent electrical properties but also be conducive to integrated fabrication processes.

This necessitates stretchable conductors with tunable conductivity, work function, smoothness, transparency, and compatibility with other materials. However, most intrinsically stretchable materials encounter technical challenges of their own. Stretchable conductive polymers, for instance, are inherently limited in stretchability. Meanwhile, stretchable composites struggle to strike a balance between conductivity and elasticity.^[^ ^4a^ ^]^ Liquid metal, renowned for its exceptional stretchability, faces challenges in patterning due to its high surface tension.^[^ ^5^ ^]^ Similarly, ionic stretchable electrodes, while offering excellent biological compatibility, are composed of hydrogels with ions or ionic liquids, inherently presenting encapsulation issues.^[^ ^6^ ^]^ Thus, the development of stretchable conductors that meet all these requirements is crucial for advancing the field of stretchable electronics.

Metalized NFs have garnered attention due to their remarkable tensile properties and electrical conductivity. By fabricating super‐long polymer fibers via electrospinning and subsequently synthesizing or depositing metal in situ, a variety of stretchable functional materials with bulk‐like electrical conductivity have been prepared.^[^ ^7^ ^]^ However, their potential for patterning has often been overlooked. Unlike other stretchable materials that struggle to integrate with traditional microfabrication processes, metalized NFs have successfully utilized micromachining technologies such as lithography and inkjet printing in production. This has enabled the realization of multifunctional soft systems, including wireless transmission systems, sensors, semiconductor and energy applications, and intelligent healthcare devices. These pioneering works implied their potential in fabricating stretchable devices with high integration density and multifunctionality.

**Figure 1:**
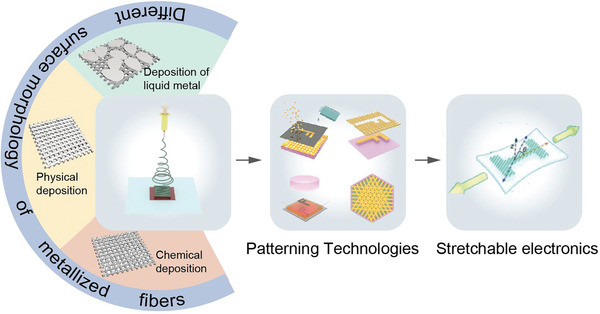
Different NF metalizing methods and patterning technologies used in stretchable electronics. First, metalized NFs with different surface morphologies are obtained by electrospinning technology and different metal deposition methods. Then, after different patterning processing, metalized films are applied in stretchable electronics.

In this review, the focus is on the development of metallized NFs within the domain of stretchable electronics (**Figure** **1**). Realizing their potential necessitates meticulous optimization of fabrication strategies. The initial section examines the fabrication process of electrospun metallized NFs, while also comparing their advantages with other conductive polymers. Subsequent sections delve into the critical aspect of patterning technologies tailored for metallized NFs, pivotal for enabling highly integrated stretchable electronics. Finally, a comprehensive analysis of state‐of‐the‐art applications in stretchable electronics based on metallized NFs is provided, alongside a summary of existing challenges and emerging trends shaping the future of NF‐based electronics.

## 2 Fundamentals of Metallized Nanofibers for Stretchable Electronics

Stretchable electronics exhibit outstanding tensile properties, enabling effective bonding to complex and uneven surfaces.^[^ ^8^ ^]^ Currently, two primary approaches are employed to achieve device stretchability. The first method, termed the structure‐based approach involves utilizing a material with a relatively high Young's modulus bonded directly to a substrate made of rubber or plastic with a low Young's modulus. Innovative designs incorporating geometric patterns and device structures, such as island‐bridge design and fractal structures, concentrate strain on the elastomeric material when the device is subjected to stress. This approach offers the advantage of integrating conventional high‐performance electronic components, although the challenge of packaging remains significant.^[^ ^3^, ^9^ ^]^ The second approach, known as the material‐based approach, constructs the device using intrinsically stretchable materials. Intrinsically stretchable electronics offer benefits such as higher device density, superior mechanical stability, and compliance, rendering them suitable for applications in medical diagnosis and flexible robotics.^[^ ^10^ ^]^

Intrinsically stretchable conductors and conductive composites are the most widely utilized strategies to realize conductors in stretchable electronics. **Table** **1** summarizes representative intrinsically stretchable materials along with their electrical and mechanical properties, as well as feasible fabrication strategies. Ionic conductors^[^ ^4^, ^11^ ^]^ have recently garnered significant attention due to their natural stretchability and conductivity. However, the inherent contradiction between conductivity and mechanical performance of ionic conductors, and the relatively low stretchability of conductive polymers, have limited their use in large‐scale applications. Similarly, finding suitable conductive fillers to satisfy both stretchability and stable electrical performance is also challenging for conductive composites. Conductive NFs have attracted considerable attention due to their extremely high aspect ratio. Among them, metallized electrospun NFs have been widely applied due to the simplicity of the preparation technologies. In the preparation process, electrospinning technology and metallization techniques play central and fundamental roles. Several important indicators of metallized NF membranes used in soft electronic devices are closely related to these two basic process parameters, including stretchability, electrical conductivity, and permeability, among others. This section delves into the basic preparation processes of metallized NFs and provides a specific analysis of the advantages of metallized NFs in soft electronics.

**Table 1:** Conductive materials used to fabricate stretchable devices.

Materials	Conductivity (S cm^−1^)/ Sheet resistance (Ohm sq.−1)	Stretchability (%)	Process	Ref.
Carbon‐based	1.9 m–2200/7‐30	50%−1050%	Mechanical cutting, printing	[101]
Metal‐based	2200–4000/1.68–11.1	100%−900%	Photolithography, nozzle printing, inkjet printing, spray printing, immerse	[48, 63, 85]
Liquid metal	7020–20600	600%−1000%	Spray printing, inject print	[5, 102]
Ionic stretchable electrodes	0.02 m–42.2 m	50%−2600%	Cast in molds	[6, 103]
Conductive polymer	24–2700	100%−250%	Immerse, photolithography	[104]

### 2.1 Electrospinning Technology for Preparing Nanofibers

In recent years, various processing techniques have been employed to prepare NFs, including drawing, template synthesis, phase separation, self‐assembly, and electrospinning^[^ ^12^ ^]^ Among these techniques, electrospinning has emerged as a mature and scalable method that has garnered significant attention in the field of stretchable electronics.^[^ ^7^, ^13^ ^]^ Electrospinning offers a high degree of tunability, allowing for the adjustment of spinning parameters such as composition, ratio of the spinning solution, and spinning time.^[^ ^14^ ^]^ This versatility enables the production of NF membranes with specialized characteristics, including biocompatibility,^[^ ^15^ ^]^ high transparency,^[^ ^16^ ^]^ super stretchability,^[^ ^17^ ^]^ and coaxial structure.^[^ ^18^ ^]^ Additionally, various surface modifications can be employed to impart different functionalities to the NF membranes.^[^ ^19^ ^]^ Furthermore, through layer‐by‐layer stacking, NF membranes can achieve natural encapsulation.^[^ ^20^ ^]^ To date, electrospun NFs have been successfully utilized in the fabrication of soft applications in tissue engineering, energy harvesting, and storage materials due to their permeability, stretchability, stability, and flexibility.^[^ ^7^ ^]^

**Figure 2:**
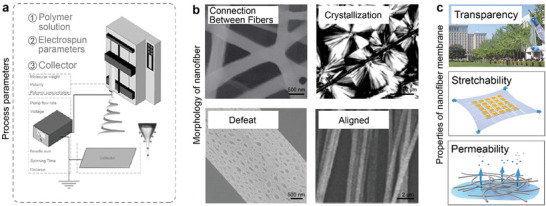
Electrospun NFs. a) Illustration of electrospun NFs and main process parameters. Reproduced with permission.^[^ ^7^ ^]^ Copyright 2019, American Chemical Society. b) Electrospun NFs with different morphological characteristics which are related to membranes’ mechanical properties. Reproduced with permission.^[^ ^105^ ^]^ Copyright 2022, Elsevier Ltd. Reproduced with permission.^[^ ^106^ ^]^ Copyright 2011, American Chemical Society. Reproduced with permission.^[^ ^107^ ^]^ Copyright 2013, American Chemical Society. Reproduced with permission.^[^ ^57^ ^]^ Copyright 2018, Wiley. c) Typical properties of NF membranes in soft electronics. Reproduced with permission.^[^ ^32^ ^]^ Copyright 2017, Wiley. Reproduced with permission.^[^ ^108^ ^]^ Copyright 2015, American Chemical Society. Reproduced with permission.^[^ ^109^ ^]^ Copyright 2022, Wiley.

As depicted in **Figure** **2**a, when a sufficiently high voltage is applied to a liquid, the charged liquid undergoes stretching and forms a liquid jet known as the Taylor cone. The charged solution jets evaporate during the ejection process, forming fibers that eventually deposit onto the collector. In the context of stretchable electronics, the mechanical properties of electrospun NF membranes need to be carefully considered. These mechanical characteristics are primarily determined by three factors: polymer parameters, electrospinning parameters, and the morphology of collectors.^[^ ^21^ ^]^ The combined influence of these factors ultimately results in variations in compatibility, viscosity between the NFs, fiber crystallinity, alignment, and orientation (Figure 2b), which in turn affect the properties of the membranes, including transparency, stretchability, and permeability (Figure 2c).^[^ ^7^ ^]^

Polymer parameters primarily encompass molecular weight, polarity, solvent ratio, solvent types, and polymer concentration. Different materials exhibit unique characteristics, rendering them suitable for various applications. For instance, poly(vinylidene fluoride) (PVDF) is widely employed in the field of nanogenerators due to its excellent piezoelectric properties.^[^ ^21^ ^]^ Guo et al. fabricated an all‐fiber hybrid piezoelectric‐enhanced triboelectric nanogenerator by electrospinning silk fibroin and PVDF NFs onto conductive fabrics, demonstrating outstanding electrical performance with a power density of 310 µW cm^2^.^[^ ^22^ ^]^

Polyvinyl alcohol (PVA), characterized by a plethora of hydroxyl groups in its molecular chains, forms a hydrogen bonding network, enhancing the transparency of the fibers. Additionally, these hydroxyl groups facilitate the formation of hydrogen bonds with various material surfaces, leading to a strong adhesive effect.^[^ ^23^ ^]^ Moreover, owing to its solubility in water, PVA NFs are commonly utilized as sacrificial layers in numerous applications. The process involves transferring devices in water to conformally adhere them onto curved surfaces.

The molecular structure of Polyurethane (PU) NFs comprises soft and hard segments. The soft segments provide elasticity and flexibility, while the hard segments offer structural stability. Therefore, PU NF membranes typically exhibit excellent mechanical properties.^[^ ^24^ ^]^ Cui's group proposed an efficient and low‐cost electronic skin based on GO‐doped PU@PEDOT composite nanofibrous, capable of detecting dynamic and static pressure, strain, and flexion. The electronic skin sensor demonstrated high pressure sensitivity (up to 20.6 kPa^−1^), a broad sensing range (1 Pa to 20 kPa), cycling stability and repeatability of over 10 000 cycles, and strain sensitivity over a wide range (up to ≈550%).^[^ ^25^ ^]^

Compared with synthetic polymer materials, silk NFs exhibit a high degree of biocompatibility, degradability, and potential for large‐scale development.^[^ ^26^ ^]^ Yin et al. reported lightweight CNT@Silk wires integrated into smart clothing, featuring electrochromism and near‐field communication. The NF film, prepared by electrospinning, was wrapped around a rotating CNT yarn in situ, demonstrating high electrical conductivity (3.1 × 10^4^ S m^−1^), good mechanical strength, flexibility, durability, and low density (2.0–7.8 × 10^4^ g m^−3^). Spider silk fibers have also been explored for soft electronics due to their mechanical robustness, biocompatibility, and biodegradability.^[^ ^27^ ^]^

Electrospinning parameters encompass pump flow rate, needle size, applied voltage, tip‐to‐collector distance, electrospinning time, and drum speed, among others. Voltage plays a fundamental role in electrospinning, with the formation of the Taylor cone occurring only when the voltage reaches a critical threshold. Generally, an increase in voltage leads to a decrease in fiber diameter, resulting in fewer spinning defects and improved mechanical properties.^[^ ^28^ ^]^ However, excessive voltage can induce jet instability and fiber defects. Flow rate is directly linked to solvent evaporation time, thereby influencing the morphology of nanofibers (NFs). Too high a feed rate can lead to insufficient solvent evaporation, increased diameter, defects, and bead formation, while too low a feed rate can cause an asymmetric Taylor cone and uneven fiber diameters. Other spinning parameters such as needle size, tip‐to‐collector distance, electrospinning time, and drum speed can also impact the quality of nanofibers. In summary, different materials necessitate the selection of distinct spinning parameters to optimize fiber membrane performance.^[^ ^12^, ^23^ ^]^

The controlled preparation of nanofibers holds great significance for realizing potential soft applications. Controllable nanofibers exhibit superior physical and chemical properties, including enhanced mechanical properties, high tensile ratio, faster charge transport, and more regular spatial structures compared to non‐woven fabrics.^[^ ^29^ ^]^ Consequently, alignment of spinning has received widespread attention as a representative of controllable preparation. Simply by designing collectors, nanofiber patterning can be achieved, as further elaborated in Section 3.1.

### 2.2 Technologies of Metallized Nanofibers

**Figure 3:**
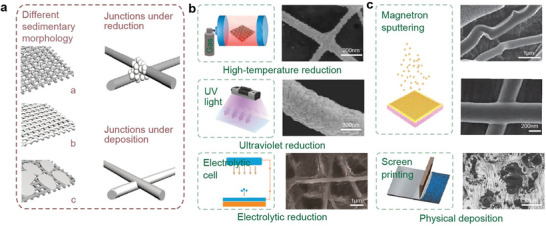
Different sedimentary morphology under different depositing methods. a) Different sedimentary morphology and junction. b) physical methods of metal deposition. Reproduced with permission.^[^ ^30^ ^]^ Copyright 2014, American Chemical Society. Reproduced with permission.^[^ ^32^ ^]^ Copyright 2017, Wiley. Reproduced with permission.^[^ ^33^ ^]^ Copyright 2015, American Chemical Society. c) metal deposition by reduction. Reproduced with permission.^[^ ^16^ ^]^ Copyright 2013, Nature. Reproduced with permission.^[^ ^15a^ ^]^ Copyright 2021, Nature.

The integration of electrospinning and metallization techniques enables the facile preparation of highly conductive and stretchable fiber networks. Essentially, metalized NFs are fabricated through in situ synthesis or deposition of metals using physical or chemical methods. As depicted in **Figure** **3**a, different deposition methods yield distinct sedimentary morphologies, thereby influencing the properties of the NF membrane such as conductivity and stretchability. Chemical deposition involves the reduction of metal ions absorbed on the NF membrane. However, this method typically results in irregular surface morphology, consequently reducing the stretchability of the membrane. Devices fabricated using this method in stretchable electronics typically exhibit a stretchability of less than 30%. On the contrary, physical deposition directly covers the metal elements through methods such as sputtering or spraying, yielding a smoother surface. Some studies have achieved stretchability of up to 900% or higher. Subsequent sections will provide a detailed overview of the preparation processes and highlight typical works employing different metallization methods.

The principle of chemical deposition involves a REDOX reaction. Initially, a precursor solution containing metal ions is electrospun onto the substrate. Subsequently, upon treatment with high temperature, the polymer components within the NFs are removed, while the metal ions are transformed into metal oxide NFs. Subsequent annealing in a hydrogen (H_2_) atmosphere reduces the metal oxide NFs into metal NFs. As depicted in Figure 3b, Cui et al. described a copper NF electrode based on this method. Precursor NFs with copper acetate dissolved in PVA are electrospun onto a substrate.^[^ ^30^ ^]^ The polymer NFs with copper precursors are then heated at 500 °C in air for 2 h to remove all polymer components, transforming the NFs into dark brown CuO NFs. During the chemical transformation from polymer fibers to CuO NFs, thermal heating melts the polymer NFs, merging the two fibers into the same identity at cross junctions points and removing any junction interface. Finally, CuO NFs are reduced into red Cu NFs after annealing in an H_2_ atmosphere at 300 °C for 1 h. This transparent electrode is transferred to a soft substrate and utilized in organic cells, exhibiting a power efficiency of 3.0%, comparable to devices made with indium tin oxide (ITO) electrodes.

Despite its simplicity, the reduction of Ag^+^ under high temperature typically requires a tube furnace, limiting the scale of the final products and restricting the choice of substrate.^[^ ^31^ ^]^ Leveraging the light‐decomposition effect of AgNO3, Ag^+^ can be reduced at room temperature and atmospheric pressure under ultraviolet irradiation. As shown in Figure 3b, Wu et al. produced flexible silver nanofiber (AgNF) electrodes for electrochromic smart windows.^[^ ^32^ ^]^ By eliminating the temperature limitation, a roll‐to‐roll process was developed for continuous production of flexible, extra‐large AgNF networks. Additionally, the electrodes exhibited a resistance of 12 Ω sq^−1^ at 95% transmittance, comparable to those of AgNF networks produced via high‐temperature sintering.

A combination of electrospinning and electroless deposition provides another cost‐effective method for producing metallized NF networks. However, as illustrated in Figure 3b, undifferentiated deposition occurs both on the NF and substrate, potentially leading to poor stretchability of the NF.^[^ ^33^ ^]^ Therefore, special treatment of the NF is necessary, such as immersion in a silver nitrate aqueous solution to obtain a seed layer on the surface, which serves as the catalytic nucleation site for subsequent electroless deposition. Cui et al. electrospun polyvinyl butyral (PVB)/SnCl_2_ NFs.^[^ ^34^ ^]^ Subsequently, a reduction of Ag^+^ occurred on the NF for Cu/Ag deposition. For both silver and copper nanowire networks, the resistance and transmittance values reached around 10 Ω sq^−1^ and 90%, respectively. This scalable process takes place at ambient temperature and pressure, opening opportunities for stretchable electronics and roll‐to‐roll large‐scale manufacturing.

As depicted in Figure 3a, metallized NFs fabricated through a reduction process commonly exhibit defective surfaces with high roughness, which can significantly influence their properties such as contact resistance and stretchability in the fabrication of stretchable electrodes.^[^ ^7^, ^35^ ^]^ Additionally, the purity of metal NFs is affected by reaction solutions and may contain impurities or oxides that impact their properties. Thermal evaporation, electron‐beam evaporation, or magnetron sputtering are typical physical deposition methods that enable uniform conformal metalizing coating of NF networks. Following the deposition processes, the polymer fibers are coated on one side, facilitating easy transfer onto various substrates. To date, a wide variety of metallized NFs, including gold, silver, copper, platinum, and aluminum NFs, have been fabricated using this method. Cui et al. demonstrated the fabrication of a transparent conducting electrode exhibiting high optoelectronic performance (sheet resistance of ≈2 Ω^−1^ at 90% transmission) through electrospinning and metal deposition.^[^ ^16^ ^]^ These metal nanotrough networks can be bent down to a radius of 2 mm or repetitively bent the film to 20 mm, 2000 times without obvious degradation in electrical conductivity. Moreover, the electrode demonstrated its practical suitability by fabricating a flexible touch‐screen device and a transparent conducting tape.

Another straightforward method to achieve physical deposition is by directly coating or printing liquid metal onto nanofibers.^[^ ^15b^ ^]^ As depicted in Figure 3c, the liquid metal distributed among the elastomeric fibers self‐organizes into a laterally mesh‐like and vertically buckled structure. Zheng's group constructed a three‐layer EGaIn electrode capable of functioning as an ECG sensor, a sweat sensor, and an electrothermal heater.^[^ ^15a^ ^]^ This vertically stacked multilayer architecture holds promise for multichannel monitoring of human physiological states and electrothermal therapy. The EGaIn‐SBS electrode also exhibited long‐cycle stability upon deformation. After more than 100 cycles of stretching to 1000% and 1800%, the electrical resistances of EGaIn‐SBS increased by only 18% and 36%, respectively.

### 2.3 Stretchable Conductors based on Metallized Nanofibers

Electrospun NFs serve as a type of conductive filler applicable to stretchable conductive composites. The fundamental principle behind preparing stretchable conductive composites involves blending conductive, non‐stretchable materials with insulating, stretchable materials. The conductivity mechanism of these composites relies on the percolation of the conductive fillers, a concept known as percolation theory. In this section, we will first introduce percolation theory, followed by an overview of commonly used conductive fillers for conductive composites. Finally, we will analyze the advantages offered by metallized NFs in this context.

#### 2.3.1 Percolation Theory

**Figure 4:**
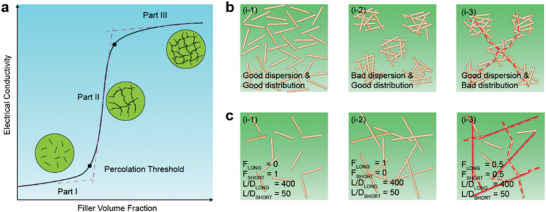
Percolation Theory. a) Description of three parts of the curve of composite conductivity versus filler volume fraction. Reproduced with permission.^[^ ^37^ ^]^ Copyright 2014, The Electrochemical Society. b) Schematic description of fillers with different dispersion and distribution conditions (i‐1): good distribution and dispersion due to random alignment which reduces the electrical conductivity in all directions; (i‐2): good distribution and bad dispersion due to agglomeration; (i‐3): effective alignment of fillers. c Schematic description of fillers with different length‐diameter ratio conditions: (i‐1): Low length‐diameter ratio fillers; (i‐2) high length‐diameter ratio fillers; (i‐3) mixture of different length‐diameter ratio fillers (easy to form conductive path). Reproduced with permission.^[^ ^37^ ^]^ Copyright 2014, The Electrochemical Society. Reproduced with permission.^[^ ^39^ ^]^ Copyright 2013, American Chemical Society.

Percolation theory provides insight into the relationship between a composite's electrical conductivity and the volume fraction of the conductive filler.^[^ ^36^ ^]^ The theory typically divides the electrical conductivity‐volume fraction curve into three distinct parts, exhibiting an “S” shape, as depicted in **Figure** **4**.^[^ ^37^ ^]^ At low filler concentrations (Figure 4a, part I), conductive particles are spaced apart, and the composite's electrical properties closely resemble those of the matrix. As the number of electronic fillers increases, conductive pathways for carrier transport form in the composite, resulting in a significant enhancement of the conductivity (Figure 4a, part II). The percolation threshold is defined as the minimum volume fraction of conductive fillers required for the composite's transformation from an insulator to a conductor. Subsequently, a plateau emerges at higher filler loadings (Figure 4a, part III). When an excess of filler is added, the crosslinking density of the elastomer network diminishes, leading to an overall decline in the mechanical performance of the elastomers, such as strain durability and mechanical toughness.^[^ ^3c^ ^]^

The percolation phenomenon is widely recognized as a complex phenomenon influenced by various independent or dependent factors. These factors include the type, aspect ratio, dispersion, distribution, surface treatment, orientation, and agglomeration of fillers, as well as the polymer type used.^[^ ^37^ ^]^ Conductive particles tend to agglomerate in the matrix, leading to poor conductivity unless long‐chain structures can be formed.^[^ ^38^ ^]^ Therefore, achieving a uniform dispersion and regular distribution of conductive particles is crucial in reducing the percolation threshold, as it allows for more conductive paths per unit volume, as illustrated in the top of Figure 4b.^[^ ^39^ ^]^ Furthermore, higher aspect ratios or mixtures of different dimensions can aid in the formation of conductive paths, as shown at the bottom of Figure 4c. Du et al. demonstrated that even with slight anisotropy, low loadings of aligned single‐wall carbon nanotube (SWNT)/poly(methyl methacrylate) (PMMA) composites can significantly increase the number of percolating clusters.^[^ ^40^ ^]^ Taipalus's group reported that the increase of carbon fiber length could reduce the percolation threshold and increase the maximum electrical conductivity.^[^ ^41^ ^]^ Moreover, Mutiso and co‐workers observed a continued reduction in sheet resistance with an increase in the fraction of high aspect ratio rods.^[^ ^39^ ^]^

#### 2.3.2 Conductive Stretchable Composite

**Figure 5:**
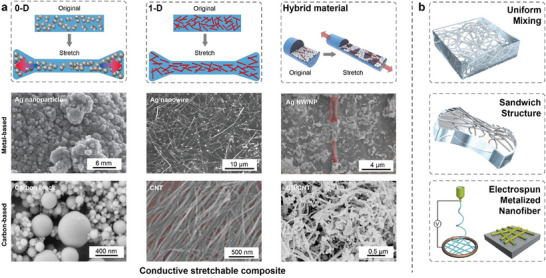
Commonly used materials and strategies in conductive composites for stretchable electronics. a) “Carbon‐based conductive filler. Carbon black. Reproduced with permission.^[^ ^38a^ ^]^ Copyright 2015, American Chemical Society. CNT. Reproduced with permission.^[^ ^42a^ ^]^ Copyright 2015, American Chemical Society. CB/CNT. Reproduced with permission.^[^ ^110^ ^]^ Copyright 2022, Elsevier Ltd. Metal‐based conductive filler. Ag nanoparticle. Reproduced with permission.^[^ ^111^ ^]^ Copyright 2012, Macmillan Publishers Limited. Ag nanowire. Reproduced with permission.^[^ ^42c^ ^]^ Copyright 2014, American Chemical Society. Ag NW/NP. Reproduced with permission.^[^ ^48^ ^]^ Copyright 2015, Wiley. b) Commonly strategies of conductive stretchable composites. Reproduced with permission.^[^ ^3c^ ^]^ Copyright 2019, Wiley. Reproduced with permission.^[^ ^16^ ^]^ Copyright 2013, Nature.

Conductive stretchable composites are formulated by integrating conductive particles with low Young's modulus into an elastic matrix. Currently, two methods are utilized to combine conductive fillers with elastic substrates: uniform mixing and sandwich structures (Figure 5b). Per percolation theory, the conductive pathways experience a significant increase when the volume fraction of fillers surpasses the percolation threshold. Consequently, a lower percolation threshold results in better stretchability for the composite.

Currently, a variety of conductive materials, including carbon‐based and metal‐based nanomaterials, have been developed for utilization in stretchable conductive electrodes or electronics.^[^ ^42^ ^]^ Carbon‐based nanomaterials, including 0D carbon black (CB), 1D CNTs, and 2D graphene, are favored not only for their moderate electrical conductivities, but also their relatively low production costs and exceptional mechanical properties.^[^ ^3^, ^43^ ^]^ Dai and colleagues have successfully developed highly stretchable and conductive electrodes by wrapping a continuous CNT thin film around pre‐stretched elastic wires to create high‐performance, stretchable wire‐shaped supercapacitors.^[^ ^44^ ^]^ Due to the high conductivity and ductility of metal material, various metal‐based fillers, such as nanoparticles, nanowires (NWs),^[^ ^45^ ^]^ and nanoflakes, have found increasing applications in stretchable conductive electrodes (**Figure** **5**a).^[^ ^38^, ^46^ ^]^ Ag NWs have shown great potential for practical use in stretchable electronics due to their high intrinsic electrical conductivity, ease of large‐scale synthesis, and low fabrication costs. Inspired by the structure of veins in a leaf or nerve systems, Soltanian et al. proposed a stretchable web of core–shell silver NFs (Ag NFs) with a lower percolation threshold, high transparency, and low sheet resistance.^[^ ^47^ ^]^ This web can also be transferred and adhered to desired substrates, offering convenience in various situations, and has been successfully utilized to manufacture numerous flexible and stretchable displays.

Recently, several strategies have been explored to reduce the percolation threshold and enhance the elastic properties of stretchable electronics. One effective method involves mixing different dimensions or types of conductive fillers. Using particles of varying sizes can significantly increase the conductive path within the elastic mixture, thereby improving the overall conductivity of the electrodes.^[^ ^48^ ^]^ Someya's group reported a printable elastic conductor with remarkable conductivity by mixing micrometer‐sized Ag flakes, fluorine rubbers, and surfactants.^[^ ^49^ ^]^ Similarly, Cui and co‐workers incorporated mesoscale metal wire into metal NW transparent conductive electrodes, reducing at least one order of magnitude in sheet resistance at a given transmittance.^[^ ^50^ ^]^ Additionally, mixing carbon‐based and metal‐based nanomaterials in hybrid configurations can dramatically enhance both conductivity and flexibility.^[^ ^38b^ ^]^ Metal‐based nanomaterials offer high conductivity, while long ultrathin nanotubes create numerous of contact junctions.^[^ ^51^ ^]^ Lee et al. proved that Ag NWs served as a backbone for the percolation networks, while the CNT mesh forms spider webs in the inter‐nanowire space of the Ag NW networks, providing local paths for electrons within the Ag NWs/CNT networks.^[^ ^52^ ^]^

On the other hand, sandwich structure can also reduce percolation threshold (Figure 5b). Some researchers fabricated conductive paths through directly depositing bulk/film on stretchable substrates.^[^ ^53^ ^]^ Typically, these conductive paths are built in horseshoe shape.^[^ ^54^ ^]^ Their performance highly depends on the properties of the stretchable substrates. However, the strain in substrate can cause an enlarged local strain in horseshoe‐shaped metal conductors. When the amount of the local strain is larger than the yielding strain of the horseshoe‐shaped metal, a plastic deformation occurs.^[^ ^55^ ^]^

Compared with other conductive fillers, electrospun metalized NFs offer numerous advantages (Figure 5b). According to percolation theory, the uniform dispersion of fillers is crucial for determining the percolation threshold. Electrospun metalized NFs naturally possess high uniformity. Besides, a natural mesh‐like structure can deform out of the plane by deflecting and twisting. Electrospun NFs’ aspect ratio is a thousand times as much as normal NWs, resulting in low percolation threshold, superior conductivity and stretchability.^[^ ^46^ ^]^ Electrospun metalized NFs also boast high flexibility, facilitating process adjustments to enhance stretchability, electrical performance, and other properties such as permeability and transparency. Moreover, their planar conducting structure presents an opportunity for leveraging mature patterning techniques compatible with traditional electronic circuits. This feature underscores their potential in constructing stretchable electronics with high‐density and high‐resolution integration.

## 3 Patterning Techniques Based on Metallized NFs

Integrating multifunctionality into stretchable electronics is becoming a tendency to meet the complexity of practical applications, which naturally puts forward a requirement for micromachining accuracy. However, the high integration of the stretchable device is not compatible with traditional lithography and still needs to overcome surface instability due to the stress coming from the rigid‐soft interface. Recently, patterning techniques based on 2D metalized NFs have emerged as a promising solution for achieving stretchable devices with high integration density and multi‐functionality. As discussed above, NFs made by electrostatic spinning possess an aspect ratio of a thousand times that of normal NWs, resulting in low percolation threshold, high transparency. Physical deposition allows for uniform conformal metalizing coating of a 2D NFs network, leading to lower sheet resistance, and excellent stretchability. These properties could help them fit with traditional patterning technologies such as inkjet printing, lithography technology, etc. On the other hand, this additive manufacturing process and heterogeneous assembly properties enable a flexible integration, bringing a chance for complex devices.^[^ ^7^ ^]^ In this section, we summarize recent developments in patterning techniques based on these 2D NFs networks, showcasing their potential for advancing the field of stretchable electronics.

### 3.1 Collector Design

Electrospinning is a versatile and superior technique used in the production and assembly of ordered, simple, or repetitive nanofibrous structures, offering significant advantages for fabricating specialized applications in tissue engineering, energy harvesting, and storage materials.^[^ ^13^ ^]^ The morphology and arrangement of the electrospun fibers are influenced by the electrostatic field generated by Coulomb interactions between the positive charges on the NFs and the negative charges on the collectors.^[^ ^56^ ^]^

**Figure 6:**
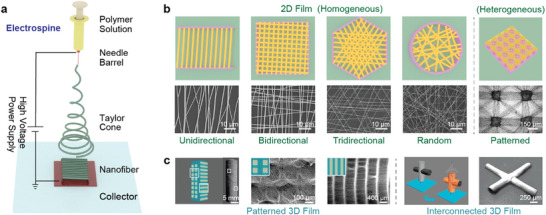
Patterning technology based on collector collection. a) Electrospinning process. b) Different schematic illustrations of collectors and corresponding SEM images of NFs. Homogeneous films. Reproduced with permission.^[^ ^57^ ^]^ Copyright 2018, Wiley. Heterogenous film. Reproduced with permission.^[^ ^58a^ ^]^ Copyright 2008, American Chemical Society. c) 3D collectors and corresponding SEM images of NFs. Reproduced with permission.^[^ ^60^ ^]^ Copyright 2008, American Chemical Society.

Patterned substrates used as collectors in electrospinning offers a means to alter the electric field and create different shapes of fibers. By using the collector with parallel edges, Cui et al. incorporated a mesoscale parallel NF in metal NF electrodes, resulting in a significant reduction in resistance by several orders of magnitude.^[^ ^50^ ^]^ Similarly, Wang and colleagues demonstrated that the electrical and mechanical properties of the metalized NFs can be further controlled by employing unidirectional, bidirectional, tri‐directional, and random collectors.^[^ ^57^ ^]^ **Figure** **6**b illustrates the scanning electron microscopy (SEM) images of these four typical NFs formed using parallel electric fields. Moreover, introducing protrusions into the collectors can lead to a regularly patterned structure. The highest density of fibers is deposited on the protrusions (representing closer distances), and additional fibers are deposited between the protrusions.^[^ ^58^ ^]^ By aligning protrusions at certain points, patterned NFs can be fabricated (Figure 6b). Yu et al. fabricated a chessboard‐like nanofibrous polyaniline/poly(vinylidene fluoride) (PANI/PVDF) membrane by using a metal grid collector. With this patterned membrane, a highly stretchable and conductive nanofibrous PANI/PVDF sensor which was capable of detecting strains up to 110% was made.^[^ ^59^ ^]^ Recently, some works have shown a resolution of 100 µm. However, as shown in Figure 6b, there are inevitably NFs deposited between the patterns. Therefore, the formed patterns are difficult to apply directly to the field of stretchable electronics.

Apart from traditional 2D nanofibrous structures, electrospinning holds the potential in the fabrication of 3D fibrous microstructures. As shown in Figure 6c, Zhang et al. fabricated interconnected tubes with removable 3D collectors.^[^ ^60^ ^]^ This novel approach offers exciting possibilities for creating tubular scaffolds in tissue engineering applications, where specific anatomical locations and biological environments require tailored designs.

### 3.2 Vapor Deposition with Mask

**Figure 7:**
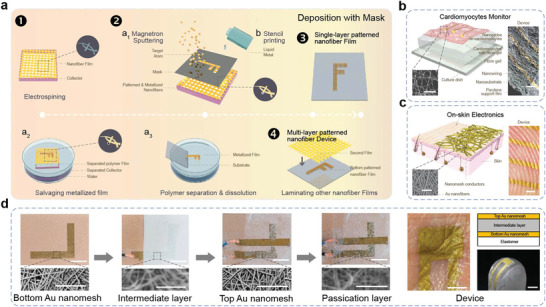
Patterning technology based on vapor deposition with a mask. a) Fabricating process by vapor deposition with a mask. The fabricating processes include electrospinning, magnetron sputtering, stencil printing, polymer separation, and dissolution, salvaging metalized film, and laminating. b) Nanomesh tactile sensor. Reproduced with permission.^[^ ^63^ ^]^ Copyright 2017, Macmillan Publishers Limited. c) Dynamically pulsing cardiomyocytes sensor. Reproduced with permission.^[^ ^64^ ^]^ Copyright 2018, Springer Nature Limited. d) Optical images, SEM images, and structure of multi‐layer NF pressure sensor. Reproduced with permission.^[^ ^65^ ^]^ Copyright 2020, American Association for the Advancement of Science.

As discussed above, recent advances in near‐field electrospinning (also known as precision electrospinning or direct‐writing electrospinning) have shown promise in controlling fiber deposition to form patterns. However, significant challenges remain in achieving precise patterning of electronics with complex structures solely through the electrospinning technique. To address these challenges, researchers have turned to vapor deposition technology, which utilizes physical and chemical processes in the gas phase to modify the composition of device surfaces and form metal or compound coatings with special properties.^[^ ^61^ ^]^ Vapor deposition is widely used in fabricating high‐precision electronic devices and large‐scale integrated circuits, making it a crucial component in traditional micro‐nano processing technology. Recently, several research groups have combined vapor deposition technology with the electrospinning technique to achieve stretchable electronics with high patterning accuracy. Using a mask during the vapor deposition process enables patterning and metallization of the NFs in a single step, simplifying the process of patterning the conductive filler layer. As shown in **Figure** **7**a, a dense NF film is initially prepared, and a certain metal layer is deposited on the device through physical vapor deposition (PVD). After depositing, a patterned conductive NF film can be easily transferred onto another substrate in a specific resolution facilitating the removal of the sacrificial layer. By repeating these steps and laminating additional NF films on the single‐layer patterned NF film, a multi‐layer NF device can be achieved.

Metalized electrospun NFs by vapor deposition with masks have proven to be highly valuable for electrode patterning due to their simple operation.^[^ ^62^ ^]^ Someya's and his team demonstrated the fabrication of a gas‐permeable stretchable sensor directly laminated onto human skin for long‐term wear without discomfort (Figure 7b).^[^ ^63^ ^]^ In this device, Au nanomesh electrodes were prepared using a shadow mask by taking electrospun polyvinyl alcohol (PVA) NFs as sacrificial supporting layers. In another work, they integrated this electrode into a biomedical electronic system for dynamic monitoring of pulsing cardiomyocytes (Figure 7c).^[^ ^64^ ^]^ This system, taking advantage of the exceptional softness of Au nanomeshes, overcame the challenge of accurately simulating traditional pulsing cardiomyocyte monitoring devices. Furthermore, as shown in Figure 7d, by integrating multilayered NF films (Au nanomesh, parylene/polyurethane nanomesh, Au nanomesh, polyurethane nanomesh), an all‐NF pressure sensor was fabricated, demonstrating comparable grip forces to those bare finger.^[^ ^65^ ^]^

The combination of stencil printing technology and mask can also realize the patterning of stretchable electrodes. Wang and co‐workers reported a stretchable sensor, and filters based on thermoplastic polyurethane membrane prepared by electrospinning and Galinstan.^[^ ^20^ ^]^ This conductive material had good stretchability (811%) and showed good stability after 30 cycles of 100%. Besides, all devices showed good air permeability, and cannot be affected by sulfuric acid, salt solutions, and artificial sweat. Another interesting method of metalizing NFs is through solution spraying process. After spraying, the NF will be soaked into the solution, where patterned (modified) parts will present a different deposition capacity of metal particle. Wang et al. prepared a PVDF fiber mat, and electrosprayed a SnCl_2_ solution through a mask.^[^ ^35^ ^]^ After mask removal and solvent evaporation, the SnCl_2_‐bearing mat was soaked in an Ag^+^ solution to reduce the Ag^+^, yielding Ag nanoparticles near the deposited SnCl_2_ and Ag nanoparticles were attached to the mat in the designed area by heating at 150 °C.

Mask currently has the highest resolution of 500 µm, and has been widely used in stretchable sensors. However, facing the future demand for higher precision (such as implantable stretchable neural electrodes), mask technology has certain limitations in resolution and/or blunt‐edge.

### 3.3 Photolithography and Etching

**Figure 8:**
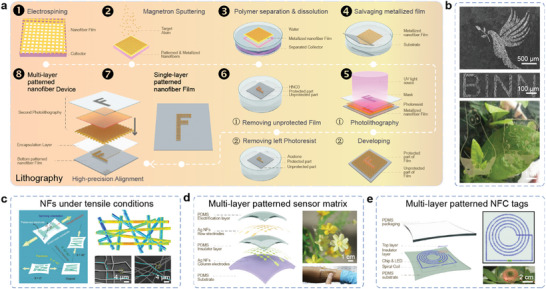
Patterning technology based on photolithography and etching. a) Schematic illustration of fabricating process by lithography. The fabricating processes include electrospinning, magnetron sputtering, polymer separation and dissolution, salvaging photolithography, and laminating. b) Digital photos of high‐precision patterned Ag NFs. Reproduced with permission.^[^ ^85^ ^]^ Copyright 2020, Macmillan Publishers Limited. c) Schematic diagram of the evolution of the unidirectional Ag NFs under different tensile conditions. Reproduced with permission.^[^ ^57^ ^]^ Copyright 2018, Wiley. Schematic diagram and SEM images of stress distribution of Ag NFs networks under tensile strain. Reproduced with permission.^[^ ^85^ ^]^ Copyright 2020, Springer Nature. d) Schematic structure and optical image of multi‐layer patterned sensor matrix for tactile imaging. Reproduced with permission.^[^ ^57^ ^]^ Copyright 2018, Wiley. e) Schematic illustration and a digital photo of the exploded view of the stretchable transparent NFC tags. Reproduced with permission.^[^ ^85^ ^]^ Copyright 2020, Springer Nature.

The photolithography process, which demonstrates excellent compatibility with electrospun NFs, has garnered significant attention for its applicability in creating various super high‐precision patterned stretchable electronics.^[^ ^66^ ^]^ The traditional lithography process facilitates the design of stretchable electronics with multilayer structures, enabling a bottom‐to‐top approach. For metalized NFs, high‐precision patterned electrodes involve a combination of transfer, lithography, and etching, effectively overcoming surface instability issue between different stretchable materials.^[^ ^67^ ^]^ This capability allows for the integration of multifunctionality into stretchable electronics through the realization of multiple overlay exposures. **Figure** **8** illustrates a schematic representation of the fabrication process. Initially, a NF thin film is fabricated on the holder using a water‐soluble material through electrospinning. Subsequently, the NFs undergo metallization by depositing a thin layer of metal on their surface via magnetron sputtering. The metalized conductive NFs are then placed on the surface of the water to dissolve polymer fiber material and subsequently transferred to the pre‐prepared substrate. After drying, a conductive thin film with a fibrous structure is obtained on the surface of the stretchable substrate through the Van der Waal's force. Next, during the photolithography process, the desired pattern is covered with a photoresist. By removing the uncovered metalized NF using a dilute nitrate solution, and subsequently eliminating the photoresist with acetone, the desired conductive pattern finally emerges. Finally, the device can be encapsulated and peeled off from the former hard substrate. For creating a multi‐layer patterned NF device, the anterior steps are repeated after laminating another NF film onto the single‐layer patterned NF film.

The latest work showed a resolution of 3.3 µm by using lithography, which is far higher than what other processes can achieve. This indicates its potential application in the future high‐precision flexible electronics. Zheng et al. designed a supersoft, stretchable, and permeable liquid metal microelectrode (µLME) based on this method.^[^ ^68^ ^]^ It had 2‐µm patterning capability, and a high density of ≈75 500 electrodes/cm^2^, and it was implanted as a neural interface for high spatiotemporal mapping and intervention of electrocorticography signals of living rats. First, Ag was patterned by photolithography on a SiO_2_ wafer, then a fibrous mat was electrospun onto the Ag micropatterns. Finally, a patterned conductive path was generated when covering the fiber mat with liquid metal. Because a selective wetting of EGaIn happened during the process due to the reactive alloying between Ag and In to form AgIn alloys. Wang and co‐workers recently reported the successful fabrication of patterned Ag NFs electrodes using this method.^[^ ^57^ ^]^ Initially, large‐scale PVA NFs are synthesized through electrospinning and subsequently coated with a thin layer of silver to form Ag NFs. Photolithography was then employed to create a series of micro‐patterned NF electrodes on a polydimethylsiloxane (PDMS) substrate, exhibiting high precision in line width, with clear edge definition for cartoon figures and letters, as shown in Figure 8b. Remarkably, these Ag NFs electrodes demonstrated excellent characteristics, including high transparency (> 70%) and low sheet resistance (1.68–11.1 Ω ^−1^). Moreover, the orientation of the Ag NFs played a critical role in achieving outstanding stretchable properties (Figure 8c). Randomly oriented Ag NFs exhibited only a 10% increase in resistance at 100% strain, whereas other orientations showed higher rise proportions. Building upon stretchable patterned Ag NFs, the research group showcased several types of sensors, such as the self‐powered triboelectric array tactile sensor, the epidermal radio frequency antenna, and the near‐field communication (NFC) tags, as depicted in Figure 8d and e. The high‐precision patterning achieved through this method presents a unique advantage over other techniques, making it highly useful for fabricating high‐precision or complex electronic devices.

### 3.4 Other Patterning Technologies

**Figure 9:**
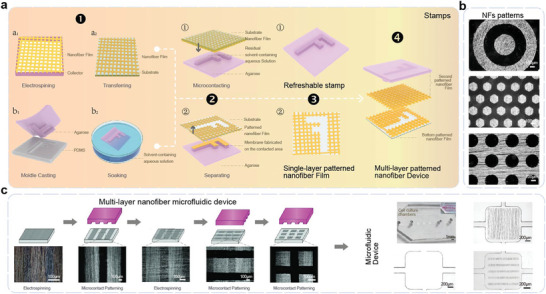
Patterning technology based on stamps. a) Fabricating process by stamps. The fabricating processes include electrospinning, molding casting, microcontact patterning, and laminating. b) SEM images of NFs with various morphologies and surface patterns. Reproduced with permission.^[^ ^70^ ^]^ c) Schematic illustrations of fabrication processes of the complex multi‐layer NF microfluidic device. Reproduced with permission.^[^ ^70^ ^]^ Copyright 2016, Wiley.

Stamp and inkjet are relatively niche methods of patterning electrospun fibers. Both methods have special requirements for the fiber materials. The latest work has proved that they can achieve a resolution of 100 and 200 µm, respectively. Nanoimprint technology is emerging as a promising alternative to traditional lithography in the realm of microelectronics and materials processing.^[^ ^69^ ^]^ Unlike conventional methods that rely on light to create patterns, nanoimprint employs mechanical means to transfer patterns, offering several advantages, such as high resolution, ease of mass production, low cost, and consistency. For electrospun NFs, transfer printing using an elastomeric stamp allows for the precise retrieval of micro‐devices from their growth substrate onto a different substrate with high‐precision patterns, as depicted in **Figure** **9**a. The process involves collecting a NF film on the substrate, while simultaneously creating an agarose hydrogel stamp by peeling off from a pre‐prepared PDMS master. The NF film is then placed onto the stamp, which has been soaked in an organic solvent‐containing aqueous solution for a certain period. The solvent at the stamp/NF interface facilitates localized coalescence of NFs, resulting in the formation of a thin membrane in the contacted area. Upon removing the NF film, patterned NFs are left on the substrate, while the stamp can be immediately refreshed for further use. By repeating this process and laminating additional films on the bottom NF film, multi‐layer patterned NF devices can be fabricated.

The transfer printing method, utilizing a micro‐structured stamp, facilitates the transfer and integration of conductive nanomaterials. Typically, an elastomer such as PDMS is used to create the stamp, which can have a patterned surface. Solvent and NF selection are crucial factors, and researchers are actively exploring suitable materials. Cao's group employed poly(l‐lactic acid) (PLLA) and 2,2,2‐trifluoroethanol (TFE) aqueous solution (TFE: H2O = 3:1, v/v) in their fabricating process.^[^ ^70^ ^]^ They initially collected a PLLA NF film and prepared agarose stamps using a PDMS master. Then these stamps were soaked in TFE solution overnight. Subsequent microcontact patterning resulted in the NF film retaining smooth and complementary patterns (compared to the patterns on the agarose stamps). This strategy allowed the realization of various single‐layer patterns, as depicted in Figure 9b. The “green” patterning method holds great potential for applications in biomedical engineering, biosensing, and lab‐on‐a‐chip systems. They successfully fabricated multi‐layer PLLA NFs and integrated them into a designated area without sacrificing overall integrity of the microfluidic device. Figure 9c showcases the manipulation and deliberate control of cell morphology and orientation within cell incubators, with or without aligned NFs. The location and ratio of oriented and unoriented cells can be precisely controlled within the same cell incubator. This technology opens up exciting possibilities for biomedical research and cell‐based applications. Similarly, Dong et al. used different materials including patterned Ag/PLLA hybrid fibers, polyethylene terephthalate/PLLA fibers, and PLLA fibers and explored their capability to manipulate biomolecule distribution and functions.^[^ ^71^ ^]^ A method provided a tool to restore spatial complexity in biomimetic matrices and would have promising applications in the field of biomedical engineering. However, despite these advancements, there are still challenges that need to be addressed during the fabricating process. One of the primary obstacles is controlling the interfacial energy between nanomaterials and the donor substrate, the receiver substrate, and among the nanomaterials themselves, to ensure the integrity and reliability of the patterned structures. Overcoming these obstacles will further advance the potential of transfer printing in high‐precision NF patterning and its applications in various fields.

Direct‐write technologies have also been considered to pattern conducting and functional inks on the fiber substrates.^[^ ^72^ ^]^ Printed patterns on a substrate need to undergo postprocessing steps such as drying and sintering to, respectively, remove solvents and binders from functional inks and generate conductive paths. Based on this, Keun Kwon and co‐workers developed PI NF‐based nerve electrode for neural signal recording.^[^ ^73^ ^]^ This electrode was fabricated simply via electrospinning and inkjet printing, while it had a high permeability, flexibility, biocompatibility, and a resistance of ≈0.31 Ω sq^−1^ for six printing repetitions. However, most NF membranes have low thermal resistances, thus could be burned out during the melting process. Therefore, some research teams tried to use liquid metals to achieve inkjet printing. Sun et al. proposed a printing system that assembled liquid metal in electrostatically driven microfluidic valves.^[^ ^74^ ^]^ However, the high roughness and porosity of NF could lead to oxidation of liquid metal, making the resistance increase during the stretching process. Moreover, this technology cannot reach high precision so far.

## 4 Recent Developments in Stretchable Electronics Based on NFs

With the rapid development of big data and the Internet of Things, our interactions with the digital world have become seamless and ubiquitous. Stretchable electronics, with its ability to achieve perfect conformal contact with living organisms, holds immense potential for the next‐generation wearable electronic technology, human‐computer interaction, and health and medical monitoring. In recent decades, significant advancements have been made in stretchable electronic devices, showcasing high performance, adaptability to biology, variable device morphology, and precise measurements. These achievements are a result of the exploration of stretchable material systems and the optimization of micromachining technology. Among the various materials used in stretchable electronics, metalized NFs stand out due to their lower percolation thresholds and compatibility with microfabrication processes. They exhibit outstanding electrical, optical, and mechanical properties, making them highly promising for applications in information technology, energy, medical treatment, national defense, and other fields. In this section, we have summarized recent developments in stretchable electronics based on NFs for different application areas. The diverse range of applications underscores the versatility and potential impact of stretchable electronics in various industries and domains.

### 4.1 Sensors

Sensor technology plays a fundamental and essential role in the development of modern science and technology, as well as in fields like modern agriculture and industrial automation. Rapid advances in soft materials and microfabrication technologies facilitate the development of stretchable sensors that are highly conformable and intimately associated with biological signal measurement. Among the hotspots in stretchable sensor research, electronic skin, electronic nose, and electronic tongue have received significant attention. These sensors encompass physical, chemical, and biological sensing capabilities, and ongoing research is dedicated to enhancing sensor sensitivity, resolution, stability, and biocompatibility.^[^ ^1^, ^75^ ^]^ The introduction of metalized NFs, together with advanced patterning techniques, has injected new vitality into sensor performance improvements, particularly in terms of transparency and conductivity.

**Figure 10:**
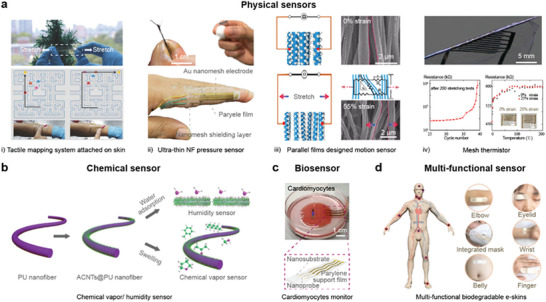
Various kinds of stretchable sensors. a) Highly stretchable tactile mapping system used in motion detection. Reproduced with permission.^[^ ^57^ ^]^ Copyright 2018, Wiley. Ultra‐thin NF pressure sensor for monitoring finger manipulation. Reproduced with permission.^[^ ^65^ ^]^ Copyright 2020, American Association for the Advancement of Science. Motion monitoring sensor with parallel films. Reproduced with permission.^[^ ^79^ ^]^ Copyright 2020, Elsevier Ltd. Fiber‐mesh polymer thermistor. Reproduced with permission.^[^ ^80^ ^]^ Copyright 2022, Wiley. b) Highly stretchable chemical vapor and humidity sensors. Reproduced with permission.^[^ ^81^ ^]^ Copyright 2019, American Chemical Society. c) Ultrasoft dynamically pulsing cardiomyocytes monitor. Reproduced with permission.^[^ ^64^ ^]^ Copyright 2018, Springer Nature. d) Whole‐body multi‐functional biodegradable e‐skins. Reproduced with permission.^[^ ^82^ ^]^ Copyright 2020, American Association for the Advancement of Science.

Physical sensors are designed to detect changes in physical stimuli,^[^ ^76^ ^]^ such as tactile, strain, acoustic, and temperature, and convert theses stimuli into electrical signals that can be measured or recorded.^[^ ^77^ ^]^ Stretchable physical sensors, particularly those based on metalized NFs with high aspect ratios, have gained popularity for biological monitoring due to their conformal nature.^[^ ^78^ ^]^ Wang et al. developed a tactile sensor using Ag NFs electrodes that performed well under high strain and enabled tactile mapping (**Figure** **10**a, i).^[^ ^57^ ^]^ These electrodes exhibited low sheet resistance (1.68–11.1 Ω^−1^), excellent stretchability, and stability even under strains exceeding 100%. To further understand the sense of natural touch, Someya's group presented an ultra‐thin NF tactile sensor for monitoring real finger manipulation (Figure 10a, ii).^[^ ^65^ ^]^ This 2 mm thick sensor was directly attached to the skin without the need for additional substrates and exhibited no significant difference in grip force compared to the bare finger. Researchers have also explored the use of electrical signal changes under deformation to create stain sensors. Similarly, Zhao's group developed a strain sensor using stencil printing.^[^ ^20^ ^]^ This method was adopted to pattern liquid metal onto a thermoplastic polyurethane membrane prepared by electrospinning. The device was then assembled layer‐by‐layer, with a stretchability of 811%, permeability of 814 L/m^2^/s, and good stability. Wang et al. fabricated a motion detector based on silver‐nanoparticle‐modified parallel polyimide NF (Figure 10a, iii).^[^ ^79^ ^]^ The sensor's resistance changed based on the overlapping or separation of polyimide films when twisted, enabling omnidirectional motion detection through a physical model of output resistance and deformation state. Moreover, stretchable temperature sensors have been developed for specialized applications in smart healthcare. Someya et al. utilized acrylate polymer and carbon NFs to create a stretchable thermistor with a three‐orders‐of‐magnitude increase in resistance within a≈2 °C temperature range (Figure 10a, iv).^[^ ^80^ ^]^ The mesh structure of NFs rendered the thermistor layer lightweight (16.5 µg cm^−2^) and transparent (more than 90% transparency in the 400–800 nm region).

Stretchable chemical sensors, biological sensors, as well as multifunctional sensors have become increasingly significant in wearable devices, biomedical engineering, and medical monitoring. To enhance the adsorption of chemical vapors, Huang's et al. demonstrated a flexible, stretchable, and conductive chemical sensor with an interconnected porous structure by decorating acidified carbon nanotube (ACNT) onto the surface of thermoplastic PU NFs (Figure 10b).^[^ ^81^ ^]^ The conductive NF could be swollen by different chemical vapors, leading to damage of the conductive network in the composite and an increase in composite resistance. For biological sensors, the challenge lies in monitoring without disrupting the natural motion of objects. Someya's group fabricated an extraordinary soft NF device to dynamically monitor pulsing cardiomyocytes (Figure 10c).^[^ ^64^ ^]^ Benefiting from the softness of the nanomeshes, NF‐attached cardiomyocytes exhibited contraction and relaxation motions without significant damage over a period of 96 h, similar to those without any substrate. Stretchable multifunctional sensors are particularly valuable for simultaneous signal detections. Heo et al. developed NF‐based electrodes for stable neural signal recording, which can be fabricated via electrospinning and inkjet printing of AgNP. Compared with traditional neuro electrodes, NF‐based electrodes showed reduced immune‐mediated pathological tissue reactions, decreased nerve atrophy, and increased long‐term biocompatibility.^[^ ^73^ ^]^ By using electrospun biodegradable material (polylactic‐co‐glycolic acid (PLGA) and PVA, Peng et al. designed a multifunctional biodegradable e‐skins to detect various physiological characteristics and movement states of the whole body, including blinking, pulsing, speaking, respiring, and major joint motion at knuckle, elbow, knee, and ankle (Figure 10d).^[^ ^82^ ^]^ The all‐NF intercross network, with Ag NW electrodes sandwiched between the top PLGA triboelectric layer and the bottom PVA substrate, contributes to the formation of a 3D micro‐to‐nano porous hierarchical structure. This structure not only provided a high specific surface area for contact electrification and pressure response but also ensured thermal‐moisture balance and wearing comfort for the skin microenvironment. Additionally, the e‐skin exhibited an antibacterial effect on E. coli (Escherichia coli) and S. aureus (Staphylococcus aureus) due to the biomedical property of Ag NWs.

### 4.2 Wireless Transmission System

Wireless transmission systems are important in electronic systems, establishing connections between devices and external systems for control, power delivery, data processing, and communication. With the development of stretchable electronics, there is a growing need for stretchability of transmission systems, including radio frequency identification (RFID) tags and communication antennas. NF‐based antennas are well‐suited for this purpose due to their stretchability, with other special properties such as transparency, tunability, and high precision, they have found extensive applications in wireless power transfer and wireless information transmission systems.

**Figure 11:**
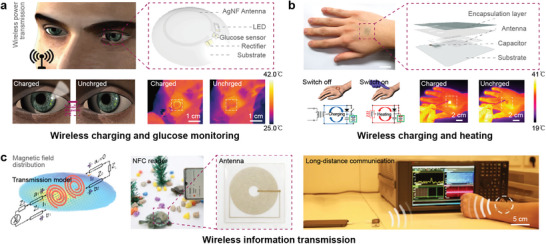
Intrinsically stretchable antennas. a) Stretchable smart contact lens. Reproduced with permission.^[^ ^83^ ^]^ Copyright 2018, American Association for the Advancement of Science. b) Operation, and heat tests of wirelessly rechargeable, skin heat patch. Reproduced with permission.^[^ ^84^ ^]^ Copyright 2020, American Chemical Society. c) Ag NFs‐based antennas used in NFC tags attached to tortoises for identification & long‐distance audio communication. Reproduced with permission.^[^ ^85^ ^]^ Copyright 2020, Springer Nature.

In wireless power transfer systems, power can be transmitted wirelessly through electromagnetic induction, providing possibilities for wireless heating and power supply. Park's team integrated a stretchable electrospun Ag NF antenna as a power receiver into a smart contact lens that could control pixels based on the glucose level in tear fluid (**Figure** **11a**).^[^ ^83^ ^]^ This antenna exhibited a low sheet resistance of 0.3 Ω sq^−1^, high transmittance of 72%, and excellent mechanical stretchability. Similarly, using a random network of CuZr nanotroughs (NTs)‐Ag NWs hybrid structure, they demonstrated a wireless heater based on the supercapacitors with a transparent, stretchable antenna for thermal therapy of skin tissue (Figure 11b).^[^ ^84^ ^]^ The hybrid network maintained good electron percolation under high deformation and had a sheet resistance of 3.0 Ω sq^−1^ at a transmittance of 91.1%.

In wireless information transmission systems, which are vital for human‐machine interaction and play essential roles in soft robotics, human healthcare monitoring, implantable medical systems etc., stretchable antennas are equally important. Wang and co‐workers exhibited a stretchable Ag NF antenna for short/long‐distance wireless information communication (Figure 11c).^[^ ^85^ ^]^ They assembled coils and NFC chips to fabricate a stretchable NFC tag that could be attached to irregular surfaces and achieve stable signal recognition. For long‐distance communication, audio signals were modulated onto the carrier wave and received by a stretchable spiral coil for auditory and visual monitoring. The tunability of the antenna was dependent on factors such as the density of Ag NFs and the shape of the electrode. The coil remained stable even after repetitive tensile testing over more than 3000 cycles at ε = 100%. Another application involved a smart contact lens and a skin‐attachable therapeutic device for wireless monitoring and therapy of chronic ocular surface inflammation.^[^ ^86^ ^]^ Field effect transistor (FET) biosensors integrated in the smart contact lens was allowed for the detection of the concentration of matrix metalloproteinase‐9 (MMP‐9) in an artificial tear solution. This information was then transmitted to a smartphone through NFC. When the signal of MMP‐9 concentration exceeded a certain threshold, the smartphone application would send a treatment command to the therapeutic device, activating the heat patch to operate at a specific therapy temperature. The Ag NF‐Ag NW antenna utilized in the device met the requirements for transparency and high‐quality factors (Q factors) of ≈7.9. Additionally, the antenna maintained stable resistance even when subjected to stretching up to 30% in tensile strain. The light, transparent, stretchable NF‐based antenna made it possible to design multi‐functional wireless tear monitoring equipment and other human‐friendly information communication systems.

### 4.3 Semiconductor Devices

Significant advancements have been made in stretchable conductive materials based on electrospun NFs, enabling the development of stretchable electrodes, sensors, and antennas. To fully equip stretchable electronic devices with active matrix and interfacing and processing circuits, it is essential to develop stretchable semiconductor materials.^[^ ^87^ ^]^ Polymer semiconductors have shown promise due to their good stretchability, but their relatively low carrier mobility remains a challenge. Electrospun NF‐based semiconductors have been proposed as a potential solution to enhance charge transfer and increase field‐effect mobility.

**Figure 12:**
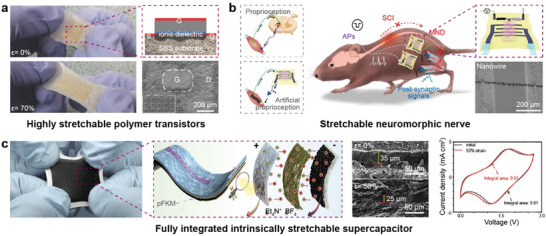
Stretchable semiconductor and energy devices. a) Stretchable polymer transistor consists of Au nanosheet electrodes, P3HT NFs as channel material, and ion‐gel as gate dielectric layer on the NF substrate. Reproduced with permission.^[^ ^88^ ^]^ Copyright 2014, Wiley. b) Stretchable neuromorphic nerve with proprioceptive feedback. Reproduced with permission.^[^ ^89^ ^]^ Copyright 2022, Springer Nature. c) SEM image, and electrochemical performance of stretchable supercapacitor with porously fibrous membrane prepared as the quasi‐solid electrolyte substrate. Reproduced with permission.^[^ ^92^ ^]^ Copyright 2021, Elsevier B.V.

FETs are critical components in traditional electronic devices, providing essential functions like switching and amplification. Stretchable FETs can to the deformation of circuits, ensuring devices function properly under strain. Researchers have demonstrated stretchable FET arrays with Au nanosheet electrodes, poly(3‐hexylthiophene) (P3HT) NFs as channel material, and an ion‐gel gate dielectric layer on the NF substrate, offering high carrier mobility and stretchability (**Figure** **12**a).^[^ ^88^ ^]^ The NF structure brought higher carrier mobility and stretchability, and the interpenetrating network structure between the ion‐gel and the porous substrate provided mechanical stability under stretching events. The average mobility of the device at 0% strain was 22 ± 0.71 cm^2^ V s, and *µ* = 18 cm^2^ V s at *ε* = 0.7. Moreover, Lee et al. demonstrated a transistor made of a single semiconducting NF as the channel material, along with an ion‐gel gate dielectric and inter‐digitated source and drain electrodes on an elastomeric substrate for a neuromorphic implant (Figure 12b).^[^ ^89^ ^]^ The NF exhibited high electrochemical surface area and low impedance (0.5 kΩ at *f*_AP_  =  1 kHz) with excellent flexibility and stretchability (100% strain). By integrated a CNT strain sensor into an artificial proprioceptor, enabling the device to bypass a broken electrophysiological signal path. This integration redirected electrophysiological signals, addressing neurological motor disorder through soft neural interfaces and stretchable electronic systems.

In addition to transistors, electrospun NFs have found applications in other stretchable electronic devices, such as photodetectors, solar cells, and supercapacitors.^[^ ^90^ ^]^ Ten and his group designed an electrospun fiber‐configured photodetector (PDs) that maintained functionality even under a strain of up to 60%. When exposed to UV light, the background conductivity of the PDs decreased rapidly and gradually returned to its initial state after being stored in the dark.^[^ ^91^ ^]^ Mu et al. reported a stretchable supercapacitor with a porously fibrous membrane, utilizing electrospun porous fluororubber (pFKM) NFs as the quasi‐solid electrolyte substrate (Figure 12c).^[^ ^92^ ^]^ This supercapacitor demonstrated a high energy density (11.8 mWh cm^−3^ at a power density of 0.0693 W cm^−3^) and maintained a capacitance retention of 90.2% after 10 000 cycles. The large surface area and high porosity of the NFs contributed to rapid liquid absorption and higher equilibrium electrolyte uptake of ≈390%, resulting in higher ionic conductivity of ≈15 mS cm^−1^.

### 4.4 Intelligent Healthcare

The advancement of society has brought forth various challenges concerning the psychological and physiological well‐being of individuals, which have underscored the significance of intelligent healthcare solutions offering smart and convenient treatments. Flexible intelligent healthcare is characterized by its large area, deformability, lightweight, and portability, enabling seamless integration into organisms through implantation, pasting, or symbiosis. This integration facilitates the profound merging of electronic systems with biological systems. In contrast, conventional bioelectronics, due to their mechanical stiffness, may lead to adverse effects on the human body and cause issues with high impedance and low signal‐to‐noise ratio. To bridge the gap between soft biological tissues and rigid bioelectronics, researchers have shifted their focus toward stretchable bioelectronics. Among the various approaches, applications based on electrospun NFs have garnered significant interest. These NFs possess essential attributes such as stretchability, lightweight, breathability, and flexibility, making them ideal for fulfilling the stringent requirements of human‐friendly devices.^[^ ^93^ ^]^

**Figure 13:**
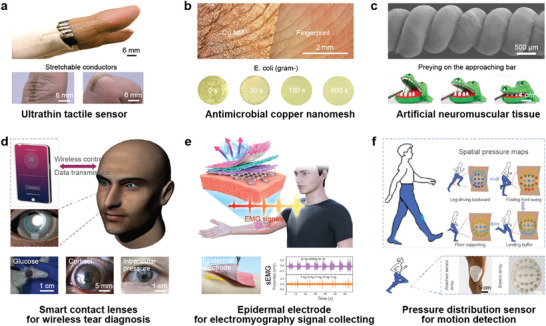
Stretchable intelligent healthcare devices. a) Nanomesh‐based pressure sensor for interference‐free monitoring of finger manipulation. Reproduced with permission.^[^ ^65^ ^]^ Copyright 2017, Macmillan Publishers Limited. b) Antimicrobial second skin using copper nanomesh. Reproduced with permission.^[^ ^95^ ^]^ Copyright 2022, PNAS. c) Fiber based artificial neuromuscular achieving the sense‐judge‐act intelligent system. Reproduced with permission.^[^ ^96^ ^]^ Copyright 2022, American Association for the Advancement of Science. d) Smart contact lenses for glucose detection. Reproduced with permission.^[^ ^83^, ^98^ ^]^ Copyright 2018, American Association for the Advancement of Science. Smart contact lenses for cortisol monitoring. Reproduced with permission. Copyright 2020, American Association for the Advancement of Science. Smart contact lenses for intraocular pressure monitoring. Reproduced with permission. Copyright 2021, Springer Nature Limited. e) Hierarchical metafabrics and surface electromyography (sEMG) signals acquired by metafabric as epidermal bioelectrodes. Reproduced with permission.^[^ ^99^ ^]^ Copyright 2022, Wiley. f) NF tactile sensor for continuous and comfortable knee joint motion monitoring. Reproduced with permission.^[^ ^100^ ^]^ Copyright 2022, Elsevier B.V.

Bioelectronics is an innovative interdisciplinary field that utilizes the principles and technologies of electronic information science to address biological problems and study biological systems. It encompasses various aspects such as information storage and transmission in biological systems, biological information acquisition, and biological information analysis. Stretchable electronics, especially those based on electrospun NFs, exhibit remarkable biocompatibility, porosity, and flexibility, making them widely applicable in biosensing, cell monitoring, and tissue engineering. Recent research has focused on developing stretchable electronic skin with multiple sensory capabilities, aiming to simulate, restore, or even replace natural human skin.^[^ ^94^ ^]^ Miyamoto et al. developed an ultrathin tactile sensation by using substrate‐free nanomesh, enabling more realistic detection of skin contact stress (**Figure** **13**a).^[^ ^65^ ^]^ This advancement has resulted in significantly reduced noise levels (<10 µV) due to low contact impedance between the device and skin. Additionally, for cell engineering, challenges arise from the inevitable contact of implanted or wearable devices with somatic cells, leading to issues such as bacterial growth during long‐term usage. In response to this, Someya's group demonstrated an ultrathin copper nanomesh with excellent antimicrobial properties to create skin protection platform (Figure 13b).^[^ ^95^ ^]^ The thin and porous nanostructure promotes biological conformality, reducing the impact of foreign bodies on skin conditions and preventing cross‐infection from harmful bacteria like E. coli and influenza virus A. Additionally, researchers are exploring the use of biocompatible and flexible NF materials based on tissue engineering principles to construct tissues or organs in vitro or in vivo. An intriguing example is the fiber‐based artificial neuromuscular reported by Dong et al. (Figure 13c),^[^ ^96^ ^]^ which emulated the touch and contraction mechanisms of snail antennae. This artificial neuromuscular fiber had a coaxial structure, obtained by wrapping a CNT fiber core in sequence with an elastomer layer, a NF network, and an MXene/CNT thin sheath, and could perceive multi‐somatosensory excitation signals, including proximity, stretch, and pressure. The introduction of the proximity‐perceiving mode allowed these fibers to make decisions on whether to actuate based on the speed of approaching objects, exhibiting an ingenious sense‐judge‐act intelligence system. Implantable devices are of interest because they can access physiological signals directly, but high demands are placed on stretchable materials. NF‐based electrodes are widely considered because they are super soft and have high permeability. Zheng's group reported a wafer‐scale neural interface on NFs using liquid metal and lithography technology.^[^ ^68^ ^]^ These liquid metal microelectrode (µLME) arrays, with chronic biocompatibility, demonstrate 2‐µm patterning capability, or a high density of ≈75500 electrodes cm^−2^. Besides, they showed super softness, electrical conductance even when stretched up to 1000% strain.

Stretchable intelligent healthcare holds the potential for innovative medical devices and cutting‐edge diagnostics in disease monitoring and treatment. Body fluid (sweat, tears, saliva) information plays an important indicative role in the real‐time monitoring of individual vital signs and disease diagnosis.^[^ ^97^ ^]^ For effective and continuous monitoring, wearable body fluid devices must be compatible for long‐term usage to timely collect and analyze pH values, glucose, cortisol, and other bodily contents. NF‐based stretchable devices fulfill these requirements with their excellent stretchability, lightweight nature, and breathability. Park's group has been actively developing smart contact lenses for wireless diagnostics, wherein the sensor and antenna materials must be transparent, stretchable, and harmless to the human body. They have utilized Ag NFs spiral antennas in various works, enabling glucose level monitoring, cortisol concentration detection, and intraocular pressure monitoring (Figure 13d).^[^ ^83^, ^98^ ^]^ In the domain of monitoring electromyogram (EMG) signals, Dong et al. introduced a hierarchically engineered stretchable, thermal‐wet comfortable, and antibacterial epidermal electrode (Figure 13e).^[^ ^99^ ^]^ This innovative electrode consists of superhydrophobic Styrene‐Ethylene‐Butylene‐Styrene (SEBS) and SEBS/PPO‐PEO‐PPO (F127) electrospun as a bilayered fabric, with hydrophilic SEBS/Thermochromic microcapsules (TMs) laminated as the top layer. The controlled reduction in fiber diameters (20.74 to 1.75 µm) and pore size (76.7 to 13.6 µm), along with the porosity‐wettability dual‐gradient, facilitates directional sweat transportation, making it ideal for long‐term bio‐signal monitoring. Additionally, for long‐time continual motion monitoring, Wang et al. demonstrated a wearable NF‐based sensor array for mapping the spatial pressure distribution of the whole knee (Figure 13f).^[^ ^100^ ^]^ The sensor boasted outstanding air (≈9.1 mm s^−1^) and moisture (≈142.69 g m^−2^ h^−1^ at 25 °C) permeability, sweat inert, thermally conductive, benefiting from the multi‐channeled porous NF network. No signs of inflammation were observed after adhering the sensor to the skin for ten days.

## 5 Conclusions

Here, we reviewed patterning technologies based on metalized NFs used in stretchable electronics. Stretchable conductors have been the subject of tremendous research efforts. New materials and novel fabrication processes have been developed to improve the stretchability, conductivity, stability, integration capabilities of stretchable conductors. However, most stretchable conductors still have issues related to instability under large mechanical deformations (buckled forms, conductive polymers), difficulty in patterning (ion conductors, conductive composites, liquid metal). It is necessary to develop new stretchable conductor materials to satisfy all these requirements. One promising approach for overcoming these issues is metalized NFs. According to the percolation theory, electrospun NFs with a super‐high ratio aspect are possible to impart electronic materials with good stretchability and conductivity. In addition, it shows highly compatible with traditional patterning technologies like mask design, lithography, and nanoimprint, contributing to the fabrication of high‐resolution and registration devices. What's more, electrospinning is a highly versatile technique and has been used as an ideal platform for fabricating NFs with biocompatibility, porosity, transparency, etc. Recent advancements in patterning techniques have propelled the integration of stretchable electronics based on electrospun NFs into various applications, including stretchable sensors, semiconductors, wireless transmission systems, and intelligent healthcare.

In recent years, many techniques and materials for metalized NFs have emerged, and different methods will affect the surface morphology, electrical performance, and stretchability of metal NFs. Reduction of electrospun NFs under high temperature limits large‐scale preparation processes. While ultraviolet reduction can proceed in room temperature, all the metallized NFs produced by reduction methods has surface defects, which will affect their conductive properties and tensile properties as well. Physical deposition through thermal evaporation, electron‐beam evaporation, magnetron sputtering can largely circumvent the problem, but the transfer process is too complicated, which brings the burden of cost and instability of device performance. Therefore, large‐scale and stable preparation of metal NFs still faces challenges.

Facing the advanced stretchable devices in the future, another irreplaceable capability of metalized NFs is their compatibility with traditional high‐precision patterning technologies such as inject printing, photolithographing, stencil printing etc. In recent years, many patterning techniques have been applied to building a diverse range of applications including stretchable sensors, semiconductors, wireless transmission systems, and intelligent healthcare systems. To be further applied in stretchable electronics, there are still several challenges need to be figured. First, the specific surface area of NFs causes metal oxidation, which will lead to electrical performance decline and shortening of life of the device. Second, the surface instability due to the stress coming from the interface which will also influences the stability and performance of the device. Third, despite the numerous patterning methods based on metal NF, it is still a great challenge to achieve the accuracy and stability of traditional high integrated electronics based on silicon. Finally, to achieve true biomedical integration, the other performances such as corrosion resistance, biocompatibility, degradability of metal NFs need to be further considered. Therefore, for metalized electrospun NFs, despite the huge potential, there is still a long way to go.

## Conflict of Interest

The authors declare no conflict of interest.

## References

[1] a) M. Amjadi , K. U. Kyung , I. Park , M. Sitti , Adv. Funct. Mater. 2016, 26, 1678;

[2] Y. S. Choi , H. Jeong , R. T. Yin , R. Avila , A. Pfenniger , J. Yoo , J. Y. Lee , A. Tzavelis , Y. J. Lee , S. W. Chen , H. S. Knight , S. Kim , H.‐Y. Ahn , G. Wickerson , A. Vázquez‐Guardado , E. Higbee‐Dempsey , B. A. Russo , M. A. Napolitano , T. J. Holleran , L. A. Razzak , A. N. Miniovich , G. Lee , B. Geist , B. Kim , S. Han , J. A. Brennan , K. Aras , S. S. Kwak , J. Kim , E. A. Waters , et al., Science 2022, 376, 1006. 35617386 10.1126/science.abm1703 PMC9282941

[3] a) J. Lee , B. L. Zambrano , J. Woo , K. Yoon , T. Lee , Adv. Mater. 2020, 32, 1902532; 10.1002/adma.201902532 31495991

[4] a) L. V. Kayser , D. J. Lipomi , Adv. Mater. 2019, 31, 1806133; 10.1002/adma.201806133 PMC6401235 30600559

[5] P. A. Lopes , D. F. Fernandes , A. F. Silva , D. G. Marques , A. T. de Almeida , C. Majidi , M. Tavakoli , ACS Appl. Mater. Interfaces 2021, 13, 14552. 33689286 10.1021/acsami.0c22206

[6] L. G. Xu , Z. K. Huang , Z. S. Deng , Z. K. Du , T. L. Sun , Z. H. Guo , K. Yue , Adv. Mater. 2021, 33, 2105306. 10.1002/adma.202105306 34647370

[7] J. J. Xue , T. Wu , Y. Q. Dai , Y. N. Xia , Chem. Rev. 2019, 119, 5298. 30916938 10.1021/acs.chemrev.8b00593 PMC6589095

[8] N. J. Matsuhisa , X. D. Chen , Z. N. Bao , T. Someya , Chem. Soc. Rev. 2019, 48, 2946. 31073551 10.1039/c8cs00814k

[9] J. T. Reeder , Z. Q. Xie , Q. S. Yang , M. H. Seo , Y. Yan , Y. J. Deng , K. R. Jinkins , S. R. Krishnan , C. Liu , S. McKay , E. Patnaude , A. Johnson , Z. C. Zhao , M. J. Kim , Y. M. Xu , I. Huang , R. Avila , C. Felicelli , E. Ray , X. Guo , W. Z. Ray , Y. G. Huang , M. R. MacEwan , J. A. Rogers , Science 2022, 377, 109. 35771907 10.1126/science.abl8532

[10] a) S. H. Wang , J. Y. Oh , J. Xu , H. Tran , Z. N. Bao , Acc. Chem. Res. 2018, 51, 1033; 29693379 10.1021/acs.accounts.8b00015

[11] J. Rivnay , S. Inal , B. A. Collins , M. Sessolo , E. Stavrinidou , X. Strakosas , C. Tassone , D. M. Delongchamp , G. G. Malliaras , Nat. Commun. 2016, 7, 11287. 27090156 10.1038/ncomms11287 PMC4838877

[12] R. Abdulhussain , A. Adebisi , B. R. Conway , K. Asare‐Addo , J. Drug Deliv. Sci. Technol. 2023, 90, 105156.

[13] Y. Wang , T. Yokota , T. Someya , NPG Asia Mater 2021, 13, 22.

[14] A. Babu , I. Aazem , R. Walden , S. Bairagi , D. M. Mulvihill , S. C. Pillai , Chem. Eng. J. 2023, 452, 139060.

[15] a) Z. J. Ma , Q. Y. Huang , Q. Xu , Q. N. Zhuang , X. Zhao , Y. H. Yang , H. Qiu , Z. L. Yang , C. Wang , Y. Chai , Z. J. Zheng , Nat. Mater. 2021, 20, 859; 33603185 10.1038/s41563-020-00902-3

[16] H. Wu , D. S. Kong , Z. C. Ruan , P. C. Hsu , S. Wang , Z. F. Yu , T. J. Carney , L. B. Hu , S. H. Fan , Y. Cui , Nat. Nanotechnol. 2013, 8, 421. 23685985 10.1038/nnano.2013.84

[17] Y. Wang , S. Lee , T. Yokota , H. Wang , Z. Jiang , J. Wang , M. Koizumi , T. Someya , Sci. Adv. 2020, 6, eabb7043. 32851182 10.1126/sciadv.abb7043 PMC7423357

[18] K. Vodsed'álková , L. Vysloužilová , L. Berezkinová , in Electrospun Materials for Tissue Engineering and Biomedical Applications (Eds.: T. Uyar , E. Kny ), Woodhead Publishing, 2017, pp. 321–336.

[19] R. Ghosh , K. Y. Pin , V. S. Reddy , W. A. D. M. Jayathilaka , D. Ji , W. Serrano‐Garcia , S. K. Bhargava , S. Ramakrishna , A. Chinnappan , Appl. Phys. Rev. 2020, 7, 041309.

[20] M. Wang , C. Ma , P. C. Uzabakiriho , X. Chen , Z. Chen , Y. Cheng , Z. Wang , G. Zhao , ACS Nano 2021, 15, 19364. 34783541 10.1021/acsnano.1c05762

[21] Y. Han , Y. Xu , S. Zhang , T. Li , S. Ramakrishna , Y. Liu , Macromol. Mater. Eng. 2020, 305, 2000230.

[22] Y. Guo , X.‐S. Zhang , Y. Wang , W. Gong , Q. Zhang , H. Wang , J. Brugger , Nano Energy 2018, 48, 152.

[23] J. S. Lee , K. H. Choi , H. D. Ghim , S. S. Kim , D. H. Chun , H. Y. Kim , W. S. Lyoo , J. Appl. Polym. Sci. 2004, 93, 1638.

[24] Y. Li , S. Xiao , Y. Luo , S. Tian , J. Tang , X. Zhang , J. Xiong , Nano Energy 2022, 104, 107884.

[25] K. Qi , J. He , H. Wang , Y. Zhou , X. You , N. Nan , W. Shao , L. Wang , B. Ding , S. Cui , ACS Appl. Mater. Interfaces 2017, 9, 42951. 28891284 10.1021/acsami.7b07935

[26] C. Wang , K. Xia , Y. Zhang , D. L. Kaplan , Acc. Chem. Res. 2019, 52, 2916. 31536330 10.1021/acs.accounts.9b00333

[27] Z. Yin , M. Jian , C. Wang , K. Xia , Z. Liu , Q. Wang , M. Zhang , H. Wang , X. Liang , X. Liang , Y. Long , X. Yu , Y. Zhang , Nano Lett. 2018, 18, 7085. 30278140 10.1021/acs.nanolett.8b03085

[28] Z. M. Huang , Y. Z. Zhang , M. Kotaki , S. Ramakrishna , Compos. Sci. Technol. 2003, 63, 2223.

[29] W.‐H. Han , M.‐Q. Wang , J.‐X. Yuan , C.‐C. Hao , C.‐J. Li , Y.–Z. Long , S. Ramakrishna , Arabian J. Chem. 2022, 15, 104193.

[30] H. Wu , L. B. Hu , M. W. Rowell , D. S. Kong , J. J. Cha , J. R. McDonough , J. Zhu , Y. Yang , M. D. McGehee , Y. Cui , Nano Lett. 2010, 10, 4242. 20738115 10.1021/nl102725k

[31] S. Lin , H. Wang , F. Wu , Q. Wang , X. Bai , D. Zu , J. Song , D. Wang , Z. Liu , Z. Li , N. Tao , K. Huang , M. Lei , B. Li , H. Wu , npj Flexible Electron. 2019, 3, 6.

[32] S. Lin , X. P. Bai , H. Y. Wang , H. L. Wang , J. N. Song , K. Huang , C. Wang , N. Wang , B. Li , M. Lei , H. Wu , Adv. Mater. 2017, 29, 1703238.

[33] Z. Liang , G. Y. Zheng , C. Liu , N. Liu , W. Y. Li , K. Yan , H. B. Yao , P.‐C. Hsu , S. Chu , Y. Cui , Nano Lett. 2015, 15, 2910. 25822282 10.1021/nl5046318

[34] P. C. Hsu , D. S. Kong , S. Wang , H. T. Wang , A. J. Welch , H. Wu , Y. Cui , J. Am. Chem. Soc. 2014, 136, 10593. 25019606 10.1021/ja505741e

[35] L. Miao , G. J. Liu , J. D. Wang , ACS Appl. Mater. Interfaces 2019, 11, 7397. 30689345 10.1021/acsami.8b20759

[36] J. Li , J. K. Kim , Compos. Sci. Technol. 2007, 67, 2114. 10.1016/j.compscitech.2007.04.016 PMC7127559 32287828

[37] R. Taherian , ECS J. Solid State Sci. Technol. 2014, 3, M26.

[38] a) E. S. Bhagavatheswaran , M. Parsekar , A. Das , H. H. Le , S. Wiessner , K. W. Stockelhuber , G. Schmaucks , G. Heinrich , J. Phys. Chem. C 2015, 119, 21723;

[39] R. M. Mutiso , M. C. Sherrott , A. R. Rathmell , B. J. Wiley , K. I. Winey , ACS Nano 2013, 7, 7654. 23930701 10.1021/nn403324t

[40] F. M. Du , J. E. Fischer , K. I. Winey , Phys. Rev. B 2005, 72, 121404(R).

[41] R. Taipalus , T. Harmia , M. Q. Zhang , K. Friedrich , Compos. Sci. Technol. 2001, 61, 801.

[42] a) S. Ryu , P. Lee , J. B. Chou , R. Xu , R. Zhao , A. J. Hart , S. G. Kim , ACS Nano 2015, 9, 5929; 26038807 10.1021/acsnano.5b00599

[43] a) M. Wang , Z. Yan , T. Wang , P. Q. Cai , S. Y. Gao , Y. Zeng , C. J. Wan , H. Wang , L. Pan , J. C. Yu , S. W. Pan , K. He , J. Lu , X. D. Chen , Nat. Electron. 2020, 3, 563;

[44] T. Chen , R. Hao , H. S. Peng , L. M. Dai , Angew. Chem., Int. Ed. 2015, 54, 618. 10.1002/anie.201409385 25404509

[45] H. W. Cui , K. Suganuma , H. Uchida , Nano Res. 2015, 8, 1604.

[46] S. De , J. N. Coleman , MRS Bull. 2011, 36, 774.

[47] S. Soltanian , R. Rahmanian , B. Gholamkhass , N. M. Kiasari , F. Ko , P. Servati , Adv. Energy Mater. 2013, 3, 1332.

[48] S. Lee , S. Shin , S. Lee , J. Seo , J. Lee , S. Son , H. J. Cho , H. Algadi , S. Al‐Sayari , D. E. Kim , T. Lee , Adv. Funct. Mater. 2015, 25, 3114.

[49] N. Matsuhisa , D. Inoue , P. Zalar , H. Jin , Y. Matsuba , A. Itoh , T. Yokota , D. Hashizume , T. Someya , Nat. Mater. 2017, 16, 834. 28504674 10.1038/nmat4904

[50] P. C. Hsu , S. Wang , H. Wu , V. K. Narasimhan , D. S. Kong , H. R. Lee , Y. Cui , Nat. Commun. 2013, 4, 2522. 24065116 10.1038/ncomms3522

[51] E. C. Garnett , W. Cai , J. J. Cha , F. Mahmood , S. T. Connor , M. G. Christoforo , Y. Cui , M. D. McGehee , M. L. Brongersma , Nat. Mater. 2012, 11, 241. 22306769 10.1038/nmat3238

[52] M. S. Lee , K. Lee , S. Y. Kim , H. Lee , J. Park , K. H. Choi , H. K. Kim , D. G. Kim , D. Y. Lee , S. Nam , J. U. Park , Nano Lett. 2013, 13, 2814. 23701320 10.1021/nl401070p

[53] D. Qi , K. Zhang , G. Tian , B. Jiang , Y. Huang , Adv. Mater. 2021, 33, 2003155. 10.1002/adma.202003155 32830370

[54] J. Jang , B. G. Hyun , S. Ji , E. Cho , B. W. An , W. H. Cheong , J.‐U. Park , NPG Asia Mater 2017, 9, e432.

[55] Y. Xiang , T. Li , Z. G. Suo , J. J. Vlassak , Appl. Phys. Lett. 2005, 87, 161910.

[56] D. Li , Y. N. Xia , Adv. Mater. 2004, 16, 1151.

[57] X. D. Wang , Y. F. Zhang , X. J. Zhang , Z. H. Huo , X. Y. Li , M. L. Que , Z. C. Peng , H. Wang , C. F. Pan , Adv. Mater. 2018, 30, 1706738. 10.1002/adma.201706738 29411908

[58] a) D. Zhang , J. Chang , Adv. Mater. 2007, 19, 3664;

[59] G. F. Yu , X. Yan , M. Yu , M. Y. Jia , W. Pan , X. X. He , W. P. Han , Z. M. Zhang , L. M. Yu , Y. Z. Long , Nanoscale 2016, 8, 2944. 26781815 10.1039/c5nr08618c

[60] D. M. Zhang , J. Chang , Nano Lett. 2008, 8, 3283. 18767890 10.1021/nl801667s

[61] L. Z. Sun , G. W. Yuan , L. B. Gao , J. Yang , M. Chhowalla , M. H. Gharahcheshmeh , K. K. Gleason , Y. S. Choi , B. H. Hong , Z. F. Liu , Nat. Rev. Methods Primers 2021, 1, 5.

[62] S. M. Park , S. Eom , D. Choi , S. J. Han , S. J. Park , D. S. Kim , Chem. Eng. J. 2018, 335, 712.

[63] A. Miyamoto , S. Lee , N. F. Cooray , S. Lee , M. Mori , N. Matsuhisa , H. Jin , L. Yoda , T. Yokota , A. Itoh , M. Sekino , H. Kawasaki , T. Ebihara , M. Amagai , T. Someya , Nat. Nanotechnol. 2017, 12, 907. 28737748 10.1038/nnano.2017.125

[64] S. Lee , D. Sasaki , D. Kim , M. Mori , T. Yokota , H. Lee , S. Park , K. Fukuda , M. Sekino , K. Matsuura , T. Shimizu , T. Someya , Nat. Nanotechnol. 2019, 14, 156. 30598525 10.1038/s41565-018-0331-8

[65] S. Lee , S. Franklin , F. A. Hassani , T. Yokota , M. O. G. Nayeem , Y. Wang , R. Leib , G. Cheng , D. W. Franklin , T. Someya , Science 2020, 370, 966. 33214278 10.1126/science.abc9735

[66] Y. Badhe , P. E. Rocha‐Flores , W. E. Voit , D. Remer , L. Costella , A. Joshi‐Imre , J. Vacuum Sci. Technol. B 2021, 39, 062801.

[67] a) C. S. Sharma , A. Sharma , M. Madou , Langmuir 2010, 26, 2218; 20070083 10.1021/la904078r

[68] Q. N. Zhuang , K. M. Yao , M. G. Wu , Z. G. Lei , F. Chen , J. Y. Li , Q. J. Mei , Y. Y. Zhou , Q. Y. Huang , X. Zhao , Y. Li , X. E. Yu , Z. J. Zheng , Sci. Adv. 2023, 9, adg8602. 10.1126/sciadv.adg8602 PMC10413659 37256954

[69] L. J. Guo , J. Phys. D: Appl. Phys. 2004, 37, R123.

[70] T. Hu , Q. T. Li , H. Dong , W. W. Xiao , L. Li , X. D. Cao , Small 2017, 13, 1602610. 10.1002/smll.201602610 27792275

[71] W. Xiao , Q. Li , H. He , W. Li , X. Cao , H. Dong , ACS Appl. Mater. Interfaces 2018, 10, 8465. 29461036 10.1021/acsami.7b18423

[72] a) Z. J. Krysiak , H. Abdolmaleki , S. Agarwala , U. Stachewicz , Polymers 2022, 14, 5043; 36433170 10.3390/polym14225043 PMC9697924

[73] D. N. Heo , H. J. Kim , Y. J. Lee , M. Heo , S. J. Lee , D. Lee , S. H. Do , S. H. Lee , I. K. Kwon , ACS Nano 2017, 11, 2961. 28196320 10.1021/acsnano.6b08390

[74] X. J. Chen , H. S. Lian , D. Y. Mo , X. Z. Ma , M. F. Gong , D. H. Sun , J. Micromech. Microeng. 2021, 31, 115005.

[75] a) Y. N. Liang , Z. X. Wu , Y. M. Wei , Q. L. Ding , M. Zilberman , K. Tao , X. Xie , J. Wu , Nano‐Micro Lett. 2022, 14, 52; 10.1007/s40820-021-00787-0 PMC8800976 35092489

[76] Z. X. Liu , C. C. Li , X. F. Zhang , H. X. Xu , Y. F. Zhou , M. W. Tian , S. J. Chen , S. Jerrams , F. L. Zhou , L. Jiang , Nano Res. 2023, 16, 7982.

[77] a) J. Lee , S. Shin , S. Lee , J. Song , S. Kang , H. Han , S. G. Kim , S. Kim , J. Seo , D. E. Kim , T. Lee , ACS Nano 2018, 12, 4259; 29617111 10.1021/acsnano.7b07795

[78] X. T. Li , H. B. Hu , T. Hua , B. G. Xu , S. X. Jiang , Nano Res. 2018, 11, 5799.

[79] Z. Y. Wang , P. Bi , Y. Yang , H. Y. Ma , Y. C. Lan , X. L. Sun , Y. Hou , H. Y. Yu , G. X. Lu , L. M. Jiang , B. P. Zhu , R. Xiong , Nano Energy 2021, 80, 105559.

[80] C. Okutani , T. Yokota , T. Someya , Adv. Sci. 2022, 9, 2202312. 10.1002/advs.202202312 PMC9596841 36057993

[81] X. W. Huang , B. Li , L. Wang , X. J. Lai , H. G. Xue , J. F. Gao , ACS Appl. Mater. Interfaces 2019, 11, 24533. 31246404 10.1021/acsami.9b04304

[82] X. Peng , K. Dong , C. Y. Ye , Y. Jiang , S. Y. Zhai , R. W. Cheng , D. Liu , X. P. Gao , J. Wang , Z. L. Wang , Sci. Adv. 2020, 6, 258. 10.1126/sciadv.aba9624 PMC7319766 32637619

[83] J. Park , J. Kim , S. Y. Kim , W. H. Cheong , J. Jang , Y. G. Park , K. Na , Y. T. Kim , J. H. Heo , C. Y. Lee , J. H. Lee , F. Bien , J. U. Park , Sci. Adv. 2018, 4, eaap9841. 29387797 10.1126/sciadv.aap9841 PMC5787380

[84] S. Lee , S. W. Kim , M. Ghidelli , H. S. An , J. Jang , A. Li Bassi , S. Y. Lee , J. U. Park , Nano Lett. 2020, 20, 4872. 32364743 10.1021/acs.nanolett.0c00869

[85] Y. F. Zhang , Z. H. Huo , X. D. Wang , X. Han , W. Q. Wu , B. S. Wan , H. Wang , J. Y. Zhai , J. Tao , C. F. Pan , Z. L. Wang , Nat. Commun. 2020, 11, 5629. 33159080 10.1038/s41467-020-19367-8 PMC7648760

[86] J. Jang , J. Kim , H. Shin , Y.‐G. Park , B. J. Joo , H. Seo , J.‐e. Won , D. W. Kim , C. Y. Lee , H. K. Kim , J.‐U. Park , Sci. Adv. 2021, 7, eabf7194. 33789904 10.1126/sciadv.abf7194 PMC8011975

[87] S. H. Wang , J. Xu , W. C. Wang , G. J. N. Wang , R. Rastak , F. Molina‐Lopez , J. W. Chung , S. M. Niu , V. R. Feig , J. Lopez , T. Lei , S. K. Kwon , Y. Kim , A. M. Foudeh , A. Ehrlich , A. Gasperini , Y. J. Yun , B. Murmann , J. B. H. Tok , Z. N. Bao , Nature 2018, 555, 83. 29466334 10.1038/nature25494

[88] M. Shin , J. H. Song , G. H. Lim , B. Lim , J. J. Park , U. Jeong , Adv. Mater. 2014, 26, 3706. 24664816 10.1002/adma.201400009

[89] Y. Lee , Y. X. Liu , D. G. Seo , J. Y. Oh , Y. Kim , J. X. Li , J. Kang , J. Kim , J. Mun , A. M. Foudeh , Z. N. Bao , T. W. Lee , Nat. Biomed. Eng. 2023, 7, 511. 35970931 10.1038/s41551-022-00918-x

[90] a) H. M. Wang , C. Y. Wang , M. Q. Jian , Q. Wang , K. L. Xia , Z. Yin , M. C. Zhang , X. P. Liang , Y. Y. Zhang , Nano Res. 2018, 11, 2347;

[91] X. C. Tan , J. M. Jian , Y. C. Qiao , T. Hirtz , G. H. Dun , Y. Z. Guo , T. R. Cui , J. D. Xu , S. R. Ji , Y. Yang , T. L. Ren , Adv. Mater. Technol. 2022, 7, 2101348.

[92] H. C. Mu , W. Q. Wang , L. F. Yang , J. Chen , X. W. Li , Y. Z. Yuan , X. H. Tian , G. C. Wang , Energy Storage Mater. 2021, 39, 130.

[93] a) W. S. Meng , M. Y. Nie , Z. Y. Liu , J. Zhou , Adv. Fiber Mater. 2021, 3, 149;

[94] Q. S. Li , C. Ding , W. Yuan , R. J. Xie , X. M. Zhou , Y. Zhao , M. Yu , Z. J. Yang , J. Sun , Q. Tian , F. Han , H. F. Li , X. P. Deng , G. L. Li , Z. Y. Liu , Adv. Fiber Mater. 2021, 3, 302.

[95] J. J. Kim , S. Ha , L. Kim , Y. Kato , Y. Wang , C. Okutani , H. Wang , C. Wang , K. Fukuda , S. Lee , T. Yokota , O. S. Kwon , T. Someya , Proc. Natl. Acad. Sci. USA 2022, 119, e2200830119. 35679344 10.1073/pnas.2200830119 PMC9214522

[96] L. Z. Dong , M. Ren , Y. L. Wang , G. H. Wang , S. Q. Zhang , X. L. Wei , J. F. He , B. Cui , Y. R. Zhao , P. P. Xu , X. N. Wang , J. T. Di , Q. W. Li , Sci. Adv. 2022, 8, eabq7703. 36383669 10.1126/sciadv.abq7703 PMC9668289

[97] J. Choi , R. Ghaffari , L. B. Baker , J. A. Rogers , Sci. Adv. 2018, 4, eaar3921. 29487915 10.1126/sciadv.aar3921 PMC5817925

[98] a) M. Ku , J. Kim , J. E. Won , W. Kang , Y. G. Park , J. Park , J. H. Lee , J. Cheon , H. H. Lee , J. U. Park , Sci. Adv. 2020, 6, eabb2891; 32923592 10.1126/sciadv.abb2891 PMC7455488

[99] J. C. Dong , Y. D. Peng , X. L. Nie , L. Li , C. Zhang , F. L. Lai , G. J. He , P. M. Ma , Q. F. Wei , Y. P. Huang , T. X. Liu , Adv. Funct. Mater. 2022, 32, 2209762.

[100] P. Wang , J. Liu , W. Yu , G. X. Li , C. Z. Meng , S. J. Guo , Nano Energy 2022, 103, 107768.

[101] a) Z. G. Yan , L. L. Wang , Y. F. Xia , R. D. Qiu , W. Q. Liu , M. Wu , Y. Zhu , S. L. Zhu , C. Y. Jia , M. M. Zhu , R. R. Cao , Z. L. Li , X. Wang , Adv. Funct. Mater. 2021, 31, 2100709;

[102] a) B. Chen , Y. D. Cao , Q. Y. Li , Z. Yan , R. Liu , Y. J. Zhao , X. Zhang , M. Y. Wu , Y. X. Qin , C. Sun , W. Yao , Z. Y. Cao , P. M. Ajayan , M. O. L. Chee , P. Dong , Z. F. Li , J. F. Shen , M. X. Ye , Nat. Commun. 2022, 13, 1206; 35260579 10.1038/s41467-022-28901-9 PMC8904466

[103] a) W. Zhang , B. H. Wu , S. T. Sun , P. Y. Wu , Nat. Commun. 2021, 12, 4082; 34215738 10.1038/s41467-021-24382-4 PMC8253733

[104] a) Q. S. Li , G. Chen , Y. J. Cui , S. B. Ji , Z. Y. Liu , C. J. Wan , Y. P. Liu , Y. H. Lu , C. X. Wang , N. Zhang , Y. Cheng , K. Q. Zhang , X. D. Chen , ACS Nano 2021, 15, 9955; 34110782 10.1021/acsnano.1c01431

[105] S. Cheon , H. Kang , H. Kim , Y. Son , J. Y. Lee , H.‐J. Shin , S.‐W. Kim , J. H. Cho , Adv. Funct. Mater. 2018, 28, 1703778.

[106] H. Li , S. Yan , Macromolecules 2011, 44, 417.

[107] P. Lu , Y. Xia , Langmuir 2013, 29, 7070. 23530752 10.1021/la400747y PMC3681866

[108] H. Park , Y. R. Jeong , J. Yun , S. Y. Hong , S. Jin , S.‐J. Lee , G. Zi , J. S. Ha , ACS Nano 2015, 9, 9974. 26381467 10.1021/acsnano.5b03510

[109] J. Oh , S. G. Jang , S. Moon , J. Kim , H. K. Park , H. S. Kim , S.‐M. Park , U. Jeong , Adv. Healthcare Mater. 2022, 11, 2102703. 10.1002/adhm.202102703 35285162

[110] J. C. Dong , D. Wang , Y. D. Peng , C. Zhang , F. L. Lai , G. J. He , P. M. Ma , W. F. Dong , Y. P. Huang , I. P. Parkin , T. X. Liu , Nano Energy 2022, 97, 107160.

[111] M. Park , J. Im , M. Shin , Y. Min , J. Park , H. Cho , S. Park , M. B. Shim , S. Jeon , D. Y. Chung , J. Bae , J. Park , U. Jeong , K. Kim , Nat. Nanotechnol. 2012, 7, 803. 23178335 10.1038/nnano.2012.206

